# Targeting Decidual CD16^+^ Immune Cells with Exosome‐Based Glucocorticoid Nanoparticles for Miscarriage

**DOI:** 10.1002/advs.202406370

**Published:** 2024-08-13

**Authors:** Linlin Wang, Zhinang Yin, Yanqiong Shen, Gang Feng, Fangfang Dai, Dongyong Yang, Zhimin Deng, Jing Yang, Ruizhi Chen, Leifeng Yang, Xian Chen, Qing Sun, Chunyu Huang, Yanxiang Cheng, Hongbing Deng, Lianghui Diao, Longfei Li, Tailang Yin

**Affiliations:** ^1^ Reproductive Medicine Center Department of Obstetrics and Gynecology Renmin Hospital of Wuhan University Wuhan University Wuhan 430072 China; ^2^ TaiKang Medical School (School of Basic Medical Sciences) Renmin Hospital of Wuhan University Wuhan University Wuhan 430072 China; ^3^ Shenzhen Key Laboratory of Reproductive Immunology for Peri‐implantation Guangdong Engineering Technology Research Center of Reproductive Immunology for Peri‐implantation Shenzhen Zhongshan Institute for Reproductive Medicine and Genetics Shenzhen Zhongshan Obstetrics and Gynecology Hospital (formerly Shenzhen Zhongshan Urology Hospital) Shenzhen 518000 China; ^4^ Xinjiang Key Laboratory of Oncology Fifth Department of Gynecologic Surgery Xinjiang Medical University Affiliated Tumor Hospital Urumqi 830000 China; ^5^ South China Institute of Environmental Sciences Ministry of Ecology and Environment Guangzhou 510655 China; ^6^ Hubei Key Laboratory of Biomass Resource Chemistry and Environmental Biotechnology School of Resource and Environmental Science Wuhan University Wuhan 430079 China

**Keywords:** engineered exosomes, glucocorticoid, immune microenvironment, maternal–fetal interface, miscarriage

## Abstract

Immune dysfunction in early pregnancy including overactivation of cytotoxic CD16^+^ NK cells and proinflammatory M1 macrophages at the maternal–fetal interface interferes with trophoblast invasion, spiral artery remodeling, and decidualization, potentially leading to miscarriage. Immunosuppressants like glucocorticoids (GCs) are used to regulate the immune microenvironment in clinical treatment, but the lack of safe and efficient tissue‐specific drug delivery systems, especially immune cell‐specific vectors, limits their widespread clinical application. Here, a previously uncharacterized delivery system is reported, termed GC‐Exo‐CD16Ab, in which GCs are loaded into purified exosomes derived from human umbilical cord mesenchymal stem cells, and subsequently decorated with antibody CD16Ab. GC‐Exo‐CD16Ab is biocompatible and has remarkable delivery efficiency toward CD16^+^ decidual natural killer (NK) cells and CD16^+^ macrophages in mice. This innovative approach effectively suppresses the cytotoxicity of decidual NK cells, inhibits M1 macrophage polarization, and regulates the decidual microenvironment, thereby enhancing placental and fetal morphology, and ultimately mitigating miscarriage risk in the abortion‐prone mice. The developed GC‐Exo‐CD16Ab provides a feasible platform for precise and tissue‐specific therapeutic strategies for miscarriage and pregnancy‐related diseases.

## Graphical abstract

Exosome‐based nanoparticles (GC‐Exo‐CD16Ab) are designed to deliver glucocorticoids (GC) specifically to decidual CD16^+^ NK cells and macrophages. This targeted delivery effectively suppresses the cytotoxicity of decidual NK cells, inhibits M1 macrophage polarization, and modulates the decidual microenvironment. These actions lead to the improved placental and fetal morphology, ultimately reducing the risk of miscarriage in abortion‐prone mice.

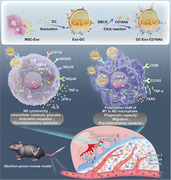

## 1 Introduction

Miscarriage, defined as pregnancy loss before 20–24 weeks of gestation, mainly occurs in early pregnancy and affects an estimated 15% of all recognized pregnancies worldwide.^[^ ^1^ ^]^ The maternal–fetal interface plays a critical role in determining pregnancy outcomes, and imbalances in immune cell populations and their functions can lead to miscarriage.^[^ ^2^ ^]^ Natural killer (NK) cells and macrophages are the most abundant immune cells at this interface, which are involved in trophoblast invasion, uterine vasculature adaptation, and fetal growth.^[^ ^3^, ^4^ ^]^ However, overactivation of cytotoxic NK cells and proinflammatory M1 macrophages in early pregnancy may lead to miscarriage.^[^ ^5^, ^6^, ^7^, ^8^ ^]^ CD16 is essential for antibody‐dependent cellular cytotoxicity (ADCC) of NK cells,^[^ ^9^ ^]^ and is also expressed by monocytes/macrophages subsets.^[^ ^10^ ^]^ CD16^+^ monocytes, but not CD16^−^ monocytes, deplete tumor‐infiltrating Tregs via ADCC, induce TNFR expression on target cells, and render them susceptible to TNFα‐mediated cell death.^[^ ^10^, ^11^ ^]^ Previous studies have revealed that a subset of cytotoxic CD16^+^ decidual NK cells significantly accumulates in the decidua of individuals experiencing miscarriage.^[^ ^5^ ^]^ These cells secrete TNF‐α and IFN‐γ, which inhibit trophoblast migration and invasion, as well as stromal cell decidualization, ultimately leading to miscarriage.^[^ ^12^, ^13^ ^]^ Moreover, cytotoxic NK cells expressing perforin and granzyme B induce apoptosis in uterine glandular cells.^[^ ^14^ ^]^ Likewise, M1 macrophages hinder trophoblast invasiveness through TNF‐α secretion and induce trophoblast apoptosis by increasing FASL expression or decreasing nitric oxide levels.^[^ ^15^ ^]^

Immunosuppressive agents such as glucocorticoids (GCs) have been used to modulate immune homeostasis in cases of miscarriage.^[^ ^16^ ^]^ However, high‐dose GCs or long‐term use of GCs can lead to various side effects, including increased susceptibility to infections, metabolic disturbances, osteoporosis, hypertension, edema, and electrolyte imbalances.^[^ ^17^ ^]^ Due to its side effects and limited dose reaching target cells, there is a demand to develop highly effective and low‐toxicity delivery systems for the treatment of pregnancy‐related diseases.^[^ ^18^ ^]^ Exosomes (Exos) are specialized cargo delivery vesicles ranging from 30 to 150 nm in size.^[^ ^19^ ^]^ They interact with target cells through direct fusion, receptor–ligand interactions, or endocytosis/phagocytosis/micropinocytosis, thereby regulating cellular biology and behavior.^[^ ^20^ ^]^ The inherent biocompatibility, transport capability, bloodstream stability, and engineerability of Exos make them potential delivery vehicles for therapeutic applications.^[^ ^21^ ^]^ For instance, M2 Exos was modified with hydrogen sulfide to promote bone regeneration through moesin‐mediated endocytosis.^[^ ^22^ ^]^ Engineered Exos enveloped with viral proteins and loaded with a polycistronic plasmid carrying three microRNAs were reported to prolong survival in a mouse model of glioblastoma.^[^ ^23^ ^]^ In addition, Exos exhibit homologous tissue‐targeting ability, which depends on the phenotype of the source cells as well as the composition and tissue origin.^[^ ^24^ ^]^ Furthermore, the placenta, including the umbilical cord, can avoid the maternal immune system, allowing its Exos to potentially not elicit inflammatory responses and evade clearance by the immune system.^[^ ^25^ ^]^ In addition, Exos derived from human umbilical cord mesenchymal stem cells (MSCs) were reported to ameliorate preeclampsia by enhancing trophoblast proliferation and migration via Notch2/TIM3/mTORC1 axis or attenuate preeclampsia via inhibition of NF‐κB/CXCL11 axis,^[^ ^26^ ^]^ which shows the potential application of MSC‐Exos in gestational diseases.

**Figure 1:**
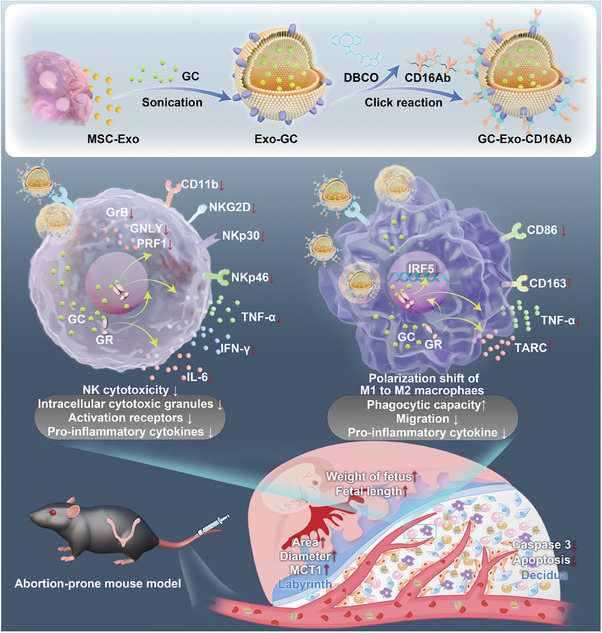
Schematic illustration of the synthesis process and immunoregulatory effect of GC‐Exo‐CD16Ab.

In this study, inspired by the potential of Exos, a novel Exo‐based drug delivery system was developed to target decidual immune cells at the maternal–fetal interface. As shown in **Figure** **1**, the engineered Exos (GC‐Exo‐CD16Ab) was constructed by encapsulating GCs into MSC‐derived Exos (MSC‐Exos) via sonication and conjugating them with CD16 antibodies (CD16Abs) through click reaction. GC‐Exo‐CD16Ab successfully targeted decidual CD16^+^ NK cells and CD16^+^ macrophages and modulated the immune microenvironment in the abortion‐prone mice, which alleviated miscarriage prominently. Our findings offer a promising and precise therapeutic strategy for addressing miscarriage and pregnancy‐related conditions, owing to its biosafety and targeted delivery capabilities.

## 2 Results

### 2.1 CD56^+^CD16^+^ NK Cells and CD68^+^CD16^+^ Macrophages Exhibit an Immune Activation Status

**Figure 2:**
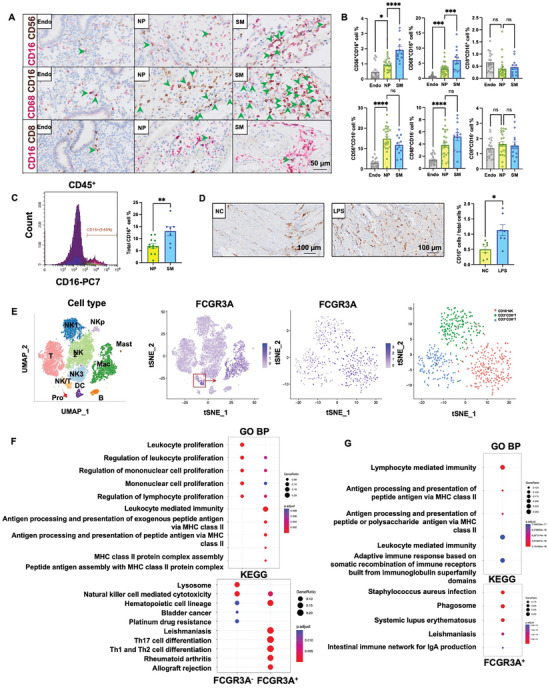
CD16^+^ NK cells and CD16^+^ macrophages are significantly dysfunctional in miscarriage decidua. A) Representative IHC images (indicated with green arrows) and B) quantitative analysis of the percentages of CD56^+^CD16^+^ NK cells, CD68^+^CD16^+^ macrophages, CD8^+^CD16^+^ T cells, and CD16^−^ immune cells in endometrium (*n* = 20) or decidua of females with NP (*n* = 26) or SM (*n* = 13) (scale bar, 50 µm). C) The percentage of CD16^+^ cells in the decidua of females with NP (*n* = 12) or SM (*n* = 7) was analyzed using FCM. D) Representative IHC images and quantitative analysis of the percentages of CD16^+^ cells in the decidua of LPS‐treated mice (scale bar, 100 µm; *n* = 7 mice per group). E) Single‐cell RNA sequencing data show the expression of CD16 (*FCGR3A*) in decidual cells, mainly expressed in NK cells and macrophages. F,G) GO BP analysis and KEGG signaling pathway analysis of the highly expressed genes of CD16^+^ NK cells versus CD16^−^ NK cells (F), and CD16^+^ macrophages versus CD16^−^ macrophages (G). Since there was no highly expressed gene in CD16^−^ macrophages (Figure S2B, Supporting Information), GO and KEGG were only analyzed for CD16^+^ macrophages. One‐way ANOVA (B), and unpaired two‐tailed *t*‐test (C, D) were used. All data are represented as mean ± standard error (SEM). Endo, healthy endometrium; NP, normal pregnancy; SM, spontaneous miscarriage. *p < 0.05, **p < 0.01, ***p < 0.001, ****p < 0.0001, and no significant differences are indicated by “n.s.”.

The percentage of CD56^+^CD16^+^ NK cells in the decidua of women with early spontaneous miscarriage (SM) was significantly higher than that of women with normal pregnancy (NP) and in normal non‐pregnant endometrium (**Figure** **2**A–C). CD56^+^CD16^−^ NK cells were also elevated in healthy decidua compared to the endometrium, but remained similar between NP and SM decidua. In contrast, the percentage of CD8^+^ T cells was consistent among groups regardless of whether CD16 positive or not (Figure 2A,B). Macrophages exhibited similar trends to NK cells (Figure 2A–C). Mice with LPS‐induced abortion also showed an increase in the CD16^+^ decidual cell population (Figure 2D). Further analysis of human decidual samples revealed that the expression of immune tolerance marker *NKG2A* in the SM group was reduced. A dysregulated cytokine profile such as anti‐inflammatory cytokines (*IL‐4, TGF‐β*) exhibited a declining trend in the SM group. Oppositely, the expressions of pro‐inflammatory cytokines (*TNF‐α, IL‐1β*) were increased in SM patients. Immune activation markers (*CD16, CD11b*) also exhibited an upward trend (Figure S1, Supporting Information). Together, these findings suggest the presence of an activated immune microenvironment in the SM decidua.

CD16 (*FCGR3A*), a classical surface marker of activated NK cells, was also expressed on decidual macrophages (Figure 2E). However, the function of CD16 on decidual macrophages is not fully understood. Compared with CD56^+^CD16^−^ NK cells, decidual CD56^+^CD16^+^ NK cells revealed a more active profile with higher expression of genes related to leukocyte‐mediated immune and inflammatory processes (Figure 2F; Figure S2A, Supporting Information). Similarly, compared with CD68^+^CD16^−^ macrophages, CD68^+^CD16^+^ macrophages appeared to be more active, exhibiting high expression levels of both pro‐ and anti‐inflammatory molecules (Figure S2B, Supporting Information). Similarly, CD68^+^CD16^+^ macrophages exhibited both pro‐ and anti‐inflammatory profiles compared to CD68^+^CD16^−^ macrophages (Figure 2G; Figure S2C–E, Supporting Information). These findings suggest that decidual CD16^+^ and CD16^−^ immune cell subsets have distinct functional roles.

### 2.2 Preparation and Characterization of GC‐Exo‐CD16Ab

**Figure 3:**
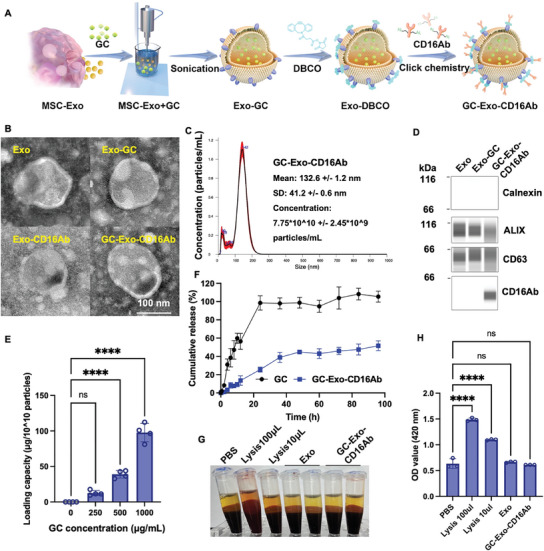
Preparation and characteristics of prepared Exos. A) Schematic representation of the synthesis of GC‐Exo‐CD16Ab. B) TEM images of Exo, Exo‐GC, Exo‐CD16Ab, and GC‐Exo‐CD16Ab (scale bar, 100 nm). C) Particle size of GC‐Exo‐CD16Ab. D) The expression of Calnexin, ALIX, CD63, and CD16Ab in the indicated Exos. E) GC‐loading capacity of GC‐Exo‐CD16Ab (*n* = 4). F) In vitro drug release profile of free GC and GC‐Exo‐CD16Ab (*n* = 3). G,H) Representative image and quantitative analysis of hemolysis of Exos (*n* = 3). One‐way ANOVA was used in (E) and (H), when compared with the controls. Data are shown as mean ± standard deviation (SD). ****p < 0.0001, and no significant differences are indicated by “n.s.”.

To address the challenges associated with conventional immunosuppressants, we developed GC‐Exo‐CD16Ab as a targeted delivery system targeting CD16^+^ cells at the maternal–fetal interface. The synthesis schematic of GC‐Exo‐CD16Ab is presented in **Figure** **3**A. Mild sonication and click reaction did not significantly affect the morphology or marker expression of Exos (Figure 3B–D). CD16Ab was successfully conjugated to Exo‐GC without inducing NK cell activation, with a loading capacity of 7.6 ± 2.2 µg/10^10^ particles (Figure 3D; Figure S3, Supporting Information). When MSC‐Exos and GCs were incubated at a 1:2 ratio, the GC‐loading capacity of GC‐Exo‐CD16Ab was determined to be 97.7 ± 12.9 µg/10^10^ particles (Figure 3E). Compared with free GC, GC‐Exo‐CD16Ab exhibited sustained GC release (Figure 3F) and no hemolysis was observed in the GC‐Exo‐CD16Ab group (Figure 3G,H).

### 2.3 GC‐Exo‐CD16Ab Exhibits High Delivery Efficiency to Decidual CD16^+^ NK Cells and CD16^+^ Macrophages

**Figure 4:**
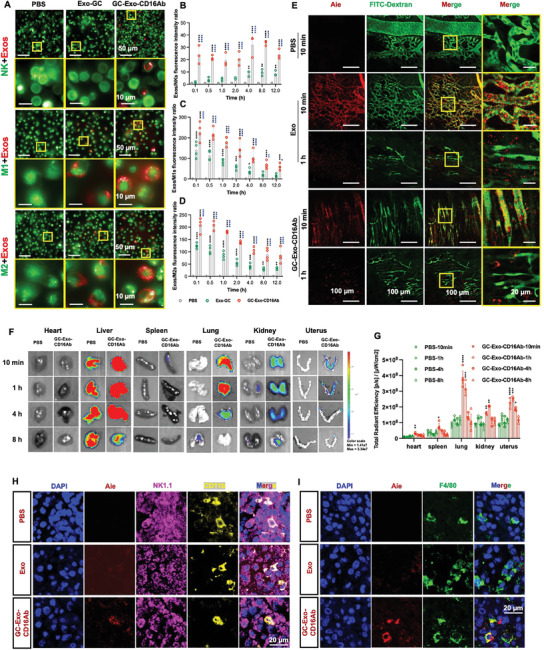
The targeting capacity of GC‐Exo‐CD16Ab in vitro and in vivo. A) Representative IF images and B–D) quantitative analysis of the Exos/NK cells, Exos/M1 macrophages, and Exos/M2 macrophages fluorescence intensity when immune cells cultured with PBS, Exo‐GC or GC‐Exo‐CD16Ab (scale bar, 50 and 10 µm). Immune cells were dyed with PKH67 and Exos were dyed with PKH26. *n* = 3–4. E) Exos dyed with AIE dye and FITC‐Dextran were injected into pregnancy mice, and the trajectory of Exos in mouse uterus was measured using two‐photon microscopy at 10 min and 1 h (scale bar, 100 and 20 µm). F,G) Distribution of GC‐Exo‐CD16Ab in different mouse organs. Mice at Gd7.5 were administered with Exos dyed with AIE dye through the tail vein. At the specified time points, the organs of mice were isolated and imaged via an IVIS imaging system. *n* = 3 mice per group. H,I) Representative IHC images of frozen uterus sections (mice at Gd7.5 and injected with AIE‐Exos for 1 h) stained with NK cell markers (NK1.1, CD11b) or macrophage marker (F4/80) (scale bar, 20 µm). One‐way ANOVA was used in (B–D, shown as mean ± SD) and (G, shown as mean ± SEM). B–D) The difference between the PBS group and the other two groups is shown with black *, and blue * indicating the difference between the Exo‐GC group and the GC‐Exo‐CD16Ab group. Data from the PBS groups in (G) were analyzed as controls. *p < 0.05, **p < 0.01, ***p < 0.001, ****p < 0.0001, and no significant differences are indicated by “n.s.”.

To access the potent targeting capacity of CD16Ab, peripheral blood NK cells and monocyte‐induced macrophages,^[^ ^27^ ^]^ both of which highly express CD16, were used in in vitro assays. Compared with Exo‐GC, GC‐Exo‐CD16Ab showed significantly higher uptake efficiency in NK cells and macrophages, with rapid uptake within minutes (**Figure** **4**A–D; Figure S4, Supporting Information). Interestingly, a time‐dependent change in the uptake of Exo‐GC was observed, possibly due to membrane fusion (Figure 4B). Compared with the PBS group, Exo‐GC uptake by M1 macrophages was dramatically enhanced at 0.1 h, and then decreased over time, possibly due to the high phagocytic capacity of macrophages and subsequent degradation of Exos.^[^ ^28^ ^]^ GC‐Exo‐CD16Ab uptake by M1 macrophages also peaked at 0.1 h and then declined (Figure 4C). M2 macrophages had the same trend as M1 macrophages (Figure 4D).

Next, in vivo tracking of FITC‐dextran and aggregation‐induced emission (AIE) dye‐labeled Exos injected into pregnant mice revealed the movement of Exos from blood vessels to local tissues. At 10 min, the majority of Exos were observed in blood vessels and then migrated to local tissues within 1 h (Figure 4E; Figure S5A,B, Supporting Information). Notably, at 1 h, Exos in mice injected with Exo were scattered in a punctate pattern within the uterine tissue, while aggregation of Exos in the GC‐Exo‐CD16Ab group presented cellular outlines (Figure 4E), indicating more efficient cellular uptake of GC‐Exo‐CD16Ab. Mice at Gd7.5 were injected with Exos to further investigate their fate in vivo, and to visualize the distribution using an in vivo imaging system. GC‐Exo‐CD16Ab mainly accumulated in the liver and lungs, followed by the uterus (Figure 4F,G; Figure S5C, Supporting Information). Except for the uterus, after intravenous administration, Exos were mostly localized in organs rich in blood vessels and organs associated with the reticuloendothelial system, such as the liver, lungs, and kidneys.^[^ ^29^ ^]^ The radiation efficiency of the uterus peaked at 1 h, subsequently decreased, and returned to baseline at 8 h. Exo and GC‐Exo‐CD16Ab had similar targeting efficiencies to the uterus at 1 h (Figure S5D–F, Supporting Information). In addition, GC‐Exo‐CD16Ab showed high delivery efficiency to the uterus at Gd9.5 and Gd11.5 (Figure S5G, Supporting Information). Frozen uterus sections (Gd7.5, injected with Exos/PBS for 1 h) demonstrate that more GC‐Exo‐CD16Ab was taken up by NK1.1^+^CD11b^+^ activated NK cells and F4/80^+^ macrophages (Figure 4H,I). The results of flow cytometry show the modification of CD16Ab significantly increased the uptake of Exos by F4/80^+^CD11b^+^ macrophages and NK1.1^+^CD11b^+^ NK cells, and decreased the delivery of Exos to CD45^−^ untargeted immune cells (Figure S6A,B, Supporting Information).

In order to evaluate the GC delivery capacity of the Exo‐GC system directly, GCs connected with Cy5.5 were used to construct the engineered Exos, and injected into the pregnancy mice at Gd7.5. Figure S6C–E (Supporting Information) shows that compared to free GCs, GCs encapsulated in Exos were better delivered to the uterus. Interestingly, free GCs were accumulated in the spleen except for the liver, lungs, and kidneys. It is well known that the spleen is an organ with a great number of immune cells, and immune cells highly express GC receptor,^[^ ^30^ ^]^ which might be the reason for the accumulation of free GCs in the spleen. Consistent with in vitro cellular uptake results, all the in vivo findings support the excellent targeting efficiency of MSC‐derived Exo‐CD16Ab to decidual activated NK cells and macrophages.

### 2.4 GC‐Exo‐CD16Ab Inhibits NK Cytotoxicity

**Figure 5:**
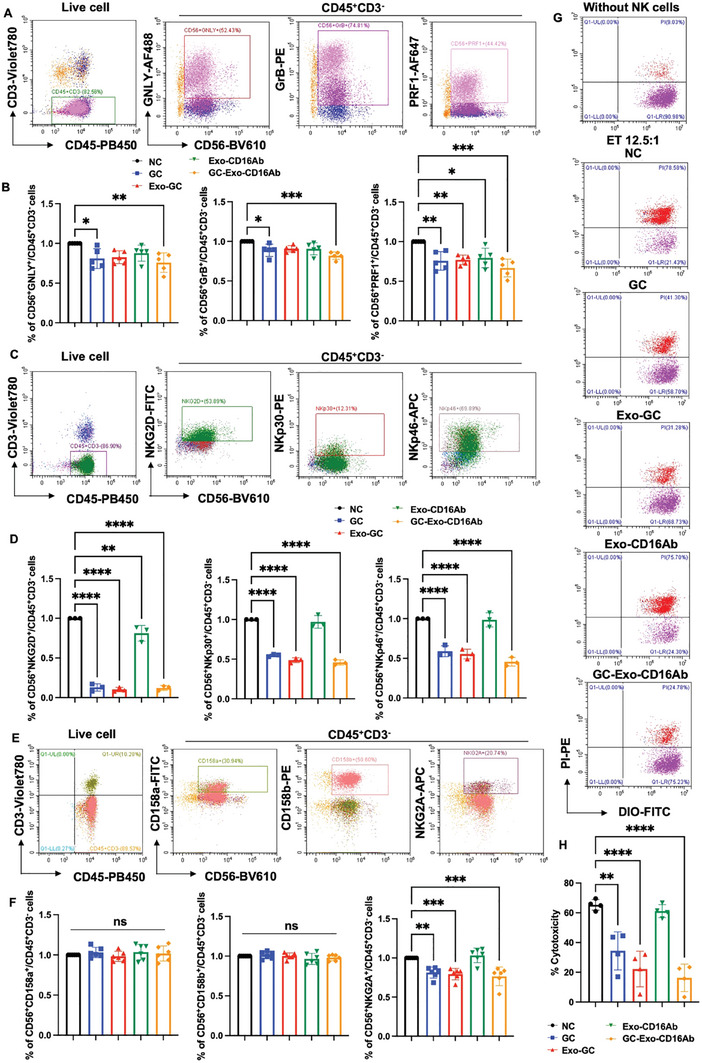
GC‐Exo‐CD16Ab inhibits NK cytotoxicity in vitro. A) Gating strategy for intracellular cytotoxic granules of NK cells. B) The percentages of CD56^+^GNLY^+^, CD56^+^CrB^+^, and CD56^+^PRF1^+^ NK cells. *n* = 5 independent experiments. C) Gating strategy for activating NK cell receptors. D) The percentages of CD56^+^NKG2D^+^, CD56^+^NKp30^+^, and CD56^+^NKp46^+^ NK cells. *n* = 3 independent experiments. E) Gating strategy for the inhibitory receptor of NK cells. F) The percentages of CD56^+^CD158a^+^, CD56^+^CD158b^+^, and CD56^+^NKG2A^+^ NK cells. *n* = 6 independent experiments. A–F) NK cells separated from peripheral blood were treated with PBS, GC, Exo‐GC, Exo‐CD16Ab, or GC‐Exo‐CD16Ab for 2 d, and then analyzed via FCM. The data of PBS groups were normalized to 1 in (B), (D), and (F). G) Representative FCM images of cytotoxicity of NK cells when treated with different GC‐Exo‐CD16Ab formulations. H) The percentages of PI^+^ K562 cells. After treatment with different GC‐Exo‐CD16Ab formulations for 2 d, NK cells were cocultured with K562 cells for 4 h at ET 12.5:1 and then analyzed via FCM. *n* = 4 independent experiments. One‐way ANOVA was used when compared with the NC group. All data are represented as mean ± SD. *p < 0.05, **p < 0.01, ***p < 0.001, ****p < 0.0001, and no significant differences are indicated by “n.s.”.

With regards to intracellular cytotoxic granules, the percentages of CD56^+^GNLY^+^, CD56^+^CrB^+^, and CD56^+^PRF1^+^ NK cells were decreased in all four treatment groups compared with the control group, indicating both GC and MSC‐Exo have immunosuppressive effects (**Figure** **5**A,B; Figure S7A, Supporting Information). GC‐Exo‐CD16Ab slightly augmented this suppressive effect compared with GC, Exo‐GC, and Exo‐CD16Ab. In terms of activating NK cell receptors, GC, Exo‐GC and GC‐Exo‐CD16Ab dramatically suppressed the percentages of CD56^+^NKG2D^+^, CD56^+^NKp30^+^, and CD56^+^NKp46^+^ NK cells (Figure 5C,D; Figure S7A, Supporting Information), and GC‐Exo‐CD16Ab showed slightly superior effects. Intriguingly, GC, Exo‐GC and GC‐Exo‐CD16Ab downregulated the inhibitory receptor NKG2A (Figure 5E,F; Figure S7A, Supporting Information). Both weakly and strongly cytotoxic NK cells have been reported to express NKG2A.^[^ ^31^ ^]^ The impact of various structures of GC‐Exo‐CD16Ab on NK cytotoxicity was consistent with their effects on activating NK cell receptors (Figure 5G,H). The CCK8 assay demonstrated that all four treatment groups were able to inhibit NK cell proliferation to some extent, among which GC‐Exo‐CD16Ab exhibited the most significant inhibitory effect (Figure S7B, Supporting Information). In addition, extracellular cytokines assay showed that GC, Exo‐GC and GC‐Exo‐CD16Ab restrained the secretion of various pro‐inflammatory cytokines such as TNF‐α, IFN‐γ, and IL‐6 (Figure S7C,D, Supporting Information). Collectively, GC‐Exo‐CD16Ab demonstrated excellent immunosuppressive effects on NK cytotoxicity.

### 2.5 GC‐Exo‐CD16Ab Promotes the Conversion of Macrophages to an Anti‐inflammatory Phenotype

**Figure 6:**
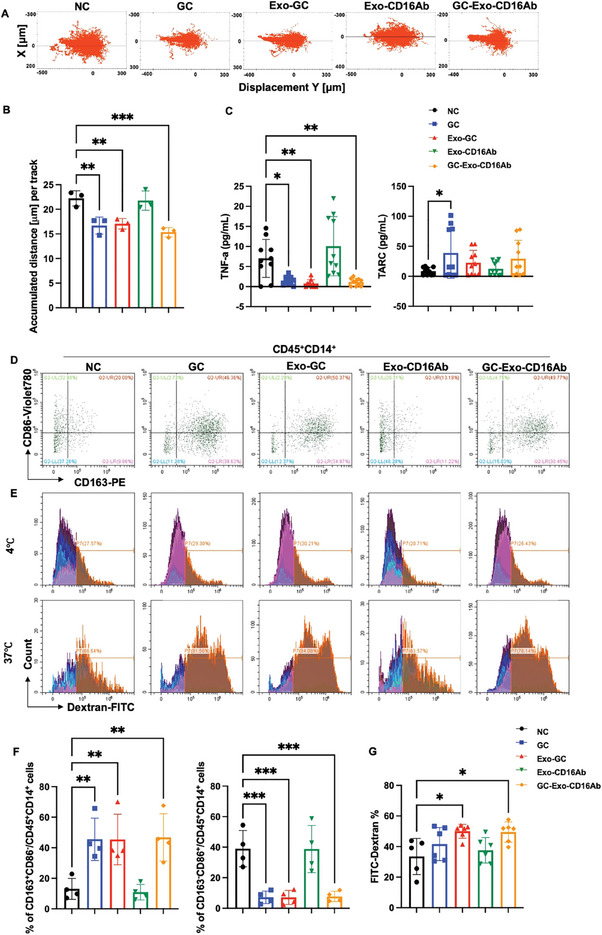
GC‐Exo‐CD16Ab promotes the polarization shift of M1 to M2 macrophages. A) Representative images and B) quantitative analysis of the M1 macrophage trajectory. Monocytes were separated from peripheral blood and induced into M1 macrophages. M1 macrophages were then treated with various GC‐Exo‐CD16Ab formulations, and the trajectory of macrophages was measured and analyzed by a High Content Analysis System for 2 d. *n* = 3 independent experiments. C) The levels of pro‐inflammatory cytokine TNF‐α and anti‐inflammatory cytokine TARC in the supernatant of M1 macrophages. *n* = 10 independent experiments. D) Representative images and F) quantitative analysis of the expression of CD163 and CD86 on M1 macrophages. M1 macrophages treated with various GC‐Exo‐CD16Ab formulations for 2 d were then analyzed by FCM. *n* = 4 independent experiments. E) Representative images and G) quantitative analysis of the percentage of FITC‐Dextran^+^ cells in total macrophages. After treatment with various GC‐Exo‐CD16Ab formulations for 2 d, M1 macrophages were cultured with FITC‐Dextran for 45 min at 37 or 4 °C and detected through FCM. *n* = 6 independent experiments. One‐way ANOVA was used when compared with the NC group. All data are represented as mean ± SD. *p < 0.05, **p < 0.01, ***p < 0.001, and no significant differences are indicated by “n.s.”.

GC, Exo‐GC and GC‐Exo‐CD16Ab significantly impaired M1 macrophage migration, proliferation, and the secretion of TNF‐α, while promoting the secretion of the anti‐inflammatory cytokine TARC (**Figure** **6**A–C; Figure S8A–C, Supporting Information). These treatments induced a polarization shift from pro‐inflammatory M1 phenotype to anti‐inflammatory M2 phenotype, with an increase in CD163^+^CD86^−^ cells and a decrease in CD163^−^CD86^+^ cells (Figure 6D,F). The phagocytic capacities of all three groups were slightly elevated (Figure 6E,G), which may be attributed to M2 polarization. GC, Exo‐GC and GC‐Exo‐CD16Ab also inhibited M2 proliferation, with GC‐Exo‐CD16Ab particularly impeding M2 migration (Figure S8D,E, Supporting Information). Given that GCs are M2 inducers, our focus was primarily on the regulation of M1 by GC‐Exo‐CD16Ab. Taken together, these results support the notion that GC‐Exo‐CD16Ab promotes macrophage polarization from pro‐inflammatory M1 phenotype to anti‐inflammatory M2 phenotype.

### 2.6 GC‐Exo‐CD16Ab Treatment Significantly Mitigates Abortion by Fostering Placental and Fetal Development

**Figure 7:**
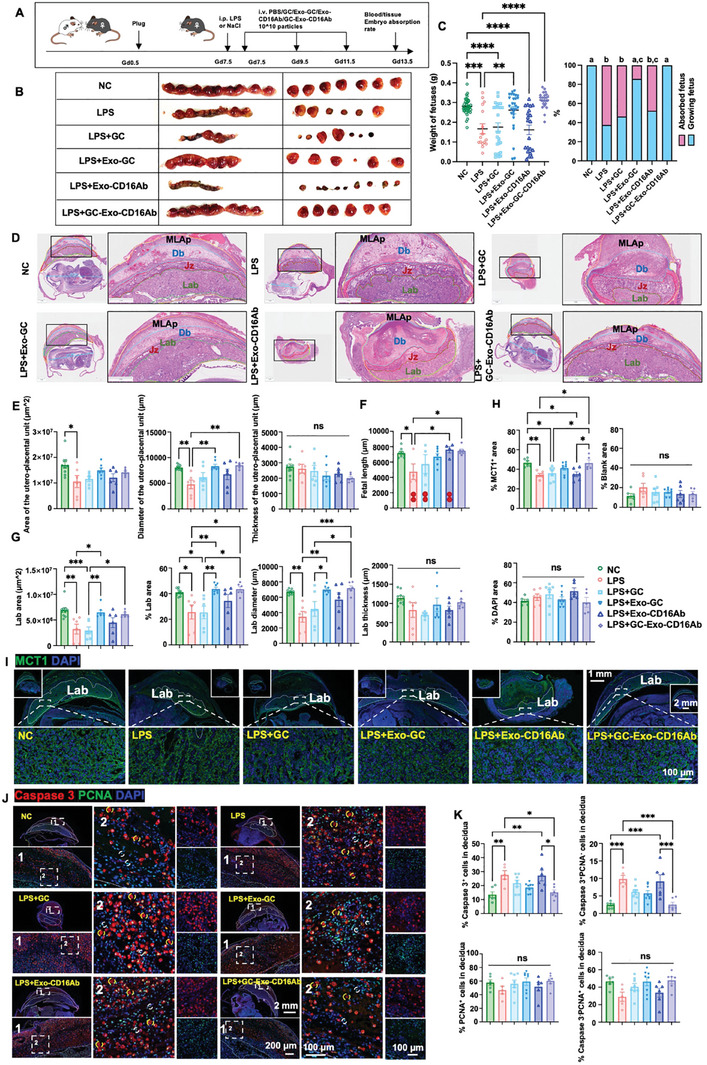
GC‐Exo‐CD16Ab promotes placental and fetal development, and alleviates abortion. A) Experimental protocol for the therapeutic treatment of LPS‐challenged abortion mice. B) Representative images of pregnancy outcomes in mice injected with various GC‐Exo‐CD16Ab formulations. C) Quantitative analysis of fetal weight and the percentages of absorbed fetuses. *n* = 3–6 mice per group. *n* = 16–39 fetuses per group. D) Representative HE images of the fetus in utero of mice treated with different GC‐Exo‐CD16Ab formulations (scale bar, 1 mm). E–G) Area, diameter, and thickness of the utero‐placental unit (E) and Lab (G), and fetal length (F) in mice injected with various GC‐Exo‐CD16Ab formulations. One red dot represents one absorbed embryo (F). *n* = 3–6 mice per group. *n* = 6–10 utero‐fetal units per group. H) Quantitative analysis and I) representative IHC images of placenta stained with MCT1 (green) in mice treated with various GC‐Exo‐CD16Ab formulations (scale bar, 2 mm, 1 mm, 100 µm). *n* = 3–6 mice per group. *n* = 5–8 utero‐fetal units per group. J) Representative IHC images and K) quantitative analysis of decidual cells stained with Caspase 3 (red) and PCNA (green) (scale bar, 2 mm, 200 µm, 100 µm, 100 µm). White dashed circles indicate Caspase 3^−^PCNA^+^ cells and yellow dashed circles indicate Caspase 3^+^PCNA^−^ cells. *n* = 3–6 mice per group. *n* = 5–8 utero‐fetal units per group. One‐way ANOVA was used. All data are represented as mean ± SEM. *p < 0.05, **p < 0.01, ***p < 0.001, ****p < 0.0001, and no significant differences are indicated by “n.s.” Letters a, b, and c were used to show statistically significant differences in the percentages of absorbed fetuses among groups via the chi‐squared test.

Based on the remarkable immunosuppressive effects of GC‐Exo‐CD16Ab in vitro, we proceeded to establish a mouse model of LPS‐induced abortion, followed by the administration of GC, Exo‐GC, Exo‐CD16Ab, and GC‐Exo‐CD16Ab to investigate their in vivo effects (**Figure** **7**A). Initially, biosafety evaluations were conducted through hematoxylin and eosin (H&E) staining, organ weight measurements, routine blood tests, and blood biochemical tests. Notably, no significant differences were observed among the groups, indicating biocompatibility of various structures of GC‐Exo‐CD16Ab (Figure S9, Supporting Information). Figure 7B shows fetuses per uterus from NC, LPS, and LPS‐challenged mouse models treated with GC, Exo‐GC, Exo‐CD16Ab, or GC‐Exo‐CD16Ab. Compared with the NC group, LPS significantly increased the number of absorbed fetuses. Exo‐GC and GC‐Exo‐CD16Ab mitigated abortion induced by LPS, while GC and Exo‐CD16Ab could not improve fetal development. Compared with the LPS group, both Exo‐GC and GC‐Exo‐CD16Ab treatments significantly promoted fetal development and decreased fetal resorption rate, with the GC‐Exo‐CD16Ab group exhibiting a better effect (Figure 7B,C). Specifically, GC‐Exo‐CD16Ab notably improved the morphology of the labyrinth (Lab) and enhanced fetal length (Figure 7D–G; Figure S10, Supporting Information). In mice with LPS‐induced abortion, the diameter of the uteroplacental unit was notably decreased but showed improvement after both Exo‐GC and GC‐Exo‐CD16Ab treatments (Figure 7E). Furthermore, Exo‐GC and GC‐Exo‐CD16Ab significantly restored Lab area and diameter, but not Lab thickness (Figure 7G). Together, GC‐Exo‐CD16Ab improved the morphology of the Lab, restored fetal length, and mitigated abortion.

Before in‐depth analysis of different formulations of GC‐Exo‐CD16Ab, we compared the effects of GC‐Exo‐CD16Ab and TGFβ‐Exo‐CD16Ab injected before and after LPS challenge on pregnancy outcomes in mice (Figure S12A, Supporting Information). TGF‐β is an anti‐inflammatory cytokine, and its reduction can lead to miscarriage due to disturbance of the immune tolerance environment and defects in placental development.^[^ ^32^ ^]^ To our surprise, both engineered Exos with biocompatibilities (Figure S11, Supporting Information) mitigated abortion, with the treatment of GC‐Exo‐CD16Ab after LPS challenge demonstrating the most pronounced effect (Figure S12B,C, Supporting Information). Both Exos primarily improved Lab morphology and restored fetal length (Figures S12D–G and S13, Supporting Information). Due to the outstanding effect of GC‐Exo‐CD16Ab, our subsequent focus was primarily on the treatment of abortion in mice after LPS challenge by GC‐Exo‐CD16Ab.

### 2.7 GC‐Exo‐CD16Ab Mitigates Structural Abnormalities of the Labyrinth, Inhibits Apoptosis in Decidual Cells, and Regulates Immune Homeostasis at the Maternal‐fetal Interface

LPS treatment significantly decreased the monocarboxylate transporter 1 (MCT1)‐positive area in the Lab, while treatment with GC‐Exo‐CD16Ab could increase the MCT1^+^ area (Figure 7H,I), indicating the positive effect of GC‐Exo‐CD16Ab on placental vascularization.^[^ ^33^ ^]^ GC‐Exo‐CD16Ab dramatically inhibited the percentages of Caspase 3^+^ cells and Caspase 3^+^PCNA^−^ cells in the decidua of abortion mice, with its effect surpassing that of GC, Exo‐GC, or Exo‐CD16Ab (Figure 7J,K). The proportions of PCNA^+^ and Caspase 3^−^PCNA^+^ cells in the decidua were consistent across groups (Figure 7J,K). Similarly, the percentages of PCNA^+^ cells in the Lab and utero‐placental regions were comparable among the 6 groups (Figure S14, Supporting Information).

**Figure 8:**
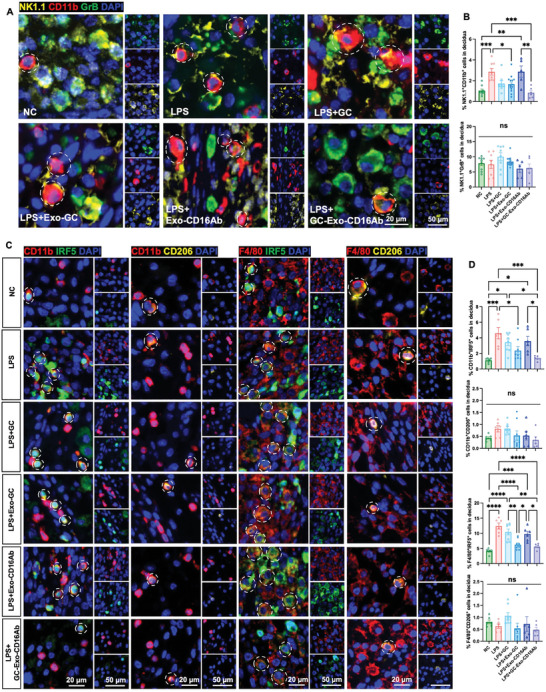
GC‐Exo‐CD16Ab regulates immune homeostasis in vivo. A) Representative IHC images and B) quantitative analysis of different NK subsets stained with NK1.1 (yellow), CD11b (red), and GrB (green) in mice treated with various GC‐Exo‐CD16Ab formulations (scale bar, 20 and 50 µm). White dashed circles indicate NK1.1^+^CD11b^+^ cells. *n* = 3–6 mice per group. *n* = 5–14 utero‐fetal units per group. C) Representative IHC images (indicated with white dashed circles) and D) quantitative analysis of CD11b^+^IRF5^+^, CD11b^+^CD206^+^, F4/80^+^IRF5^+^, and F4/80^+^CD206^+^ macrophages stained with CD11b/F4/80 (red), IRF5 (green), and CD206 (yellow) (scale bar, 20 and 50 µm). *n* = 3–6 mice per group. *n* = 6–8 utero‐fetal units per group. One‐way ANOVA was used. All data are represented as mean ± SEM. *p < 0.05, **p < 0.01, ***p < 0.001, ****p < 0.0001, and no significant differences are indicated by “n.s.”.

Given that the use of CD16Ab may interfere with the detection of CD16, CD11b, which also serves as a marker for toxic NK subsets, was utilized for the analysis of NK cell subsets.^[^ ^34^ ^]^ The proportion of NK1.1^+^CD11b^+^ cells was increased in the decidua of LPS‐treated mice, while GC‐Exo‐CD16Ab decreased their proportion (**Figure** **8**A,B). Furthermore, the immunosuppressive effect of GC‐Exo‐CD16Ab on NK1.1^+^CD11b^+^ cells was greater than that of GC, Exo‐GC or Exo‐CD16Ab. Nevertheless, the percentage of NK1.1^+^GrB^+^ cells remained unchanged among the 6 groups (Figure 8A,B), possibly due to the widespread expression of GrB in decidual NK cells, and not only in the toxic subpopulation.^[^ ^35^ ^]^ The immunosuppressive effects of Exos on CD11b^+^IFR5^+^ and F4/80^+^IRF5^+^ M1 cells were similar to their effects on NK1.1^+^CD11b^+^ cells (Figure 8C,D), while the proportions of CD206^+^ M2 cells remained consistent among groups (Figure 8C,D). Summarily, the results indicate that GC‐Exo‐CD16Ab inhibits the activation of toxic NK cells and M1 cells in vivo. Moreover, in addition to the treatment of GC‐Exo‐CD16Ab in abortion mice, administration of GC‐Exo‐CD16Ab before LPS treatment and the treatment of TGFβ‐Exo‐CD16Ab in LPS‐challenged mice showed a certain degree of suppression of decidual cell apoptosis and regulated immune homeostasis in vivo (Figures S15–S17, Supporting Information). These findings suggest that engineered MSC‐Exo represents an efficient drug delivery system for the prevention and treatment of pregnancy‐related diseases.

To further analyze how GC‐Exo‐CD16Ab modulates immune cell function and the specific pathways involved, mRNA sequencing of the mouse placenta was performed. Compared with the control group, LPS treatment upregulated immune responses related pathways, including IL‐17 and TNF signaling pathway, cytokine–cytokine receptor interaction, chemokine signaling pathway, phagosome, and complement and coagulation cascades (Figure S18A,B, Supporting Information). Treatment with GC‐Exo‐CD16Ab inhibited the above signaling pathways. On the other hand, LPS treatment inhibited biological processes like regionalization, pattern specification process, and organ development, while GC‐Exo‐CD16Ab mitigated the phenomenon (Figure S18A,C, Supporting Information). The results of Figure S18D,E (Supporting Information) further supported the above findings.

## 3 Discussion

A successful pregnancy requires precisely timed adaptations by the maternal immune system dubbed the “immune clock of pregnancy.”^[^ ^36^ ^]^ The first trimester of pregnancy is characterized by a robust increase in innate immune activity that promotes blastocyst implantation and placental development. Moreover, early pregnancy is also a state of increased vulnerability to vertically transmitted pathogens. The second trimester is marked by the establishment of an immunosuppressive environment that promotes fetal tolerance and growth. Finally, the period leading up to labor and parturition involves the reinstatement of an inflammatory milieu triggering childbirth. A cellular immunological imbalance may lead to adverse pregnancy outcomes, such as spontaneous miscarriage.^[^ ^37^ ^]^ NK cells (70%) and macrophages (20%–25%) are the two most abundant immune cells at the maternal–fetal interface.^[^ ^38^ ^]^ Single‐cell investigations of early miscarriage have suggested a pro‐inflammatory phenotype of NK cells and macrophages in the decidua.^[^ ^5^, ^6^ ^]^ Moreover, decidual NK cells with increased cytotoxicity would lead to early miscarriage.^[^ ^7^ ^]^ Decidual M1 macrophage polarization via mitochondrial dysfunction resulted in early miscarriage, while inhibiting STAT1‐mediated M1 macrophage polarization ameliorated miscarriage.^[^ ^8^ ^]^ Our study revealed significant accumulation and activation of CD16^+^ NK cells and CD16^+^ macrophages in the decidua of women with early SM (Figure 2). The overactivation of these cytotoxic and pro‐inflammatory immune cells in early pregnancy creates a hostile microenvironment that impedes critical processes such as trophoblast invasion, placental development, and fetal growth, ultimately leading to miscarriage.^[^ ^13^, ^39^ ^]^

Currently, clinical management of miscarriage often involves immunosuppressive drugs such as GCs. However, systemic administration of these drugs has limited therapeutic efficacy at the maternal‐fetal interface.^[^ ^16^ ^]^ To address the problem, we developed GC‐Exo‐CD16Ab, a novel Exo‐based drug delivery platform designed to precisely target and modulate the activity of CD16^+^ immune cells within the decidua. This strategy leverages the inherent advantages of MSC‐Exos, including excellent biocompatibility for the fetus, immune‐evasion capabilities, immunotherapeutic efficacy, and the potential for tissue‐specific homing.^[^ ^13^, ^15^, ^40^, ^41^ ^]^ By encapsulating GCs within MSC‐Exos and conjugating CD16Abs to their surface, we aimed to achieve enhanced targeting efficiency, controlled drug release, and synergistic immunomodulatory effects.

GC‐Exo‐CD16Ab exhibits potent immunosuppressive effects in vitro. Peripheral blood NK cells highly expressing CD16 were used in this study. GC‐Exo‐CD16Ab significantly inhibited the proliferation, cytotoxicity, the expression of intracellular cytotoxic granules and activating receptors, as well as pro‐inflammatory cytokine secretion of NK cells (Figure 5; Figure S7, Supporting Information). The effect of GC‐Exo‐CD16Ab surpassed that of GC and Exo‐GC, suggesting a synergistic effect of GC, Exo, and CD16Ab. Various MSC‐Exos have been reported to impede NK cell proliferation by inhibiting the NK cell cycle staying in the G0/G1 phase.^[^ ^42^ ^]^ Furthermore, the presence of thrombospondin 1 on fetal liver MSC‐derived Exos activates TGFβ through conformational changes in latency‐associated peptides, induces downstream TGFβ/Smad2/3 signaling in NK cells, and ultimately inhibits their proliferation, activation, and cytotoxicity.^[^ ^43^ ^]^ In addition, NK cells lack phagocytic capacity and interact with Exos mainly through membrane fusion or receptor‐mediated interactions.^[^ ^44^ ^]^ Therefore, the modification of Exos by CD16Ab dramatically increased the uptake of Exos by CD16^+^ NK cells in vitro (Figure 4A,B), and enhanced the immunoregulatory effect of GC‐Exo‐CD16Ab on NK cells (Figure S19A,B, Supporting Information). In terms of macrophages, CD14^+^CD16^+^ decidual macrophages with enhanced phagocytosis expressed high levels of CD163 and CD86, as well as both pro‐ and anti‐inflammatory cytokines (Figure 2G; Figure S2B–E, Supporting Information), consistent with previous studies. CD16^+^ monocytes expressed both CD163 and CD80,^[^ ^45^ ^]^ and both M1 and M2 macrophages have been reported to express CD16.^[^ ^46^ ^]^ GCs act as M2 macrophage inducers with a more pronounced effect on M1 than M2 (Figure 6A,B; Figure S8A,D–E, Supporting Information),^[^ ^47^ ^]^ prompting further investigation into the functions of GC‐Exo‐CD16Ab using M1 macrophages. GC, Exo‐GC and GC‐Exo‐CD16Ab had similar effects on the functions of M1(Figure 6; Figure S8, Supporting Information), indicating GC plays a major role in the regulation of M1. Even without CD16Ab modification, macrophages exhibited excellent Exos uptake capabilities (Figure 4C), which may contribute to the observed results.

In this study, engineered Exo GC‐Exo‐CD16Ab was innovatively utilized to achieve notable therapeutic effects in suppressing the development of abortion in vivo (Figures 7 and 8; Figures S9–S17, Supporting Information). The engineered Exo‐GC delivered GCs to the uterus and greatly enhanced pregnancy outcomes compared with systemic GC treatment. CD16Abs further promoted the precise delivery of GCs to decidual NK cells and macrophages (Figure 4E–I; Figure S6A,B, Supporting Information) and mitigated abortion. Compared with Exo‐GC, GC‐Exo‐CD16Ab significantly increased fetal weight and decreased the percentages of Caspase 3^+^PCNA^−^ cells and NK1.1^+^CD11b^+^ cytotoxic NK cells in vivo (Figure S19C–E, Supporting Information). Therefore, if considering clinical translation, GC‐Exo‐CD16Ab should be applied to attempt to improve clinical effects even though the production of Exo‐GC is simpler. In many cases, engineered Exos are designed to achieve targeted drug delivery and controlled release, ultimately resulting in favorable therapeutic outcomes. For instance, in mouse models, Exo delivery of an NF‐κB inhibitor delayed LPS‐induced preterm birth and modulated fetal immune cell signatures.^[^ ^48^ ^]^ HIF‐1α‐overexpressed Exos, when administered via the tail vein of rats, facilitated cardioprotection by upregulating pro‐angiogenic factors and enhancing neovascularization.^[^ ^49^ ^]^ Besides, treatment with GC‐Exo‐CD16Ab yielded superior results compared with treatment or prevention with TGFβ‐Exo‐CD16Ab. TGF‐β is a multifunctional cytokine that not only regulates immune responses but also induces epithelial‐mesenchymal transition and extracellular matrix deposition, potentially leading to fibrotic diseases.^[^ ^50^ ^]^ Its role in pregnancy remains incompletely understood, which may contribute to the observed results. Furthermore, embryo implantation necessitates a pro‐inflammatory microenvironment.^[^ ^51^ ^]^ Therefore, the use of GC or TGF‐β during this critical period may be detrimental to embryo implantation and fetal growth.

GC‐Exo‐CD16Ab promoted pregnancy outcomes, including amelioration of structural abnormality of Lab and increased fetal weight and length, due to its modulation of immune homeostasis. The majority of CD16^+^ NK cells express CD11b, and the expression of CD11b on NK cells signifies their maturation and activation.^[^ ^3^ ^]^ The transcription factor interferon regulatory factor 5 (IRF5) drives macrophages towards a pro‐inflammatory state.^[^ ^52^ ^]^ GC‐Exo‐CD16Ab significantly inhibited CD11b^+^ NK cells and IRF5^+^ macrophages in the decidua of abortion mice (Figure 8). It has been documented that fetal resorption induced by poly(I:C) can be prevented by depleting decidual NK cells, neutralizing TNF‐α, or blocking NKG2D.^[^ ^4^ ^]^ Additionally, ADCC targeting anti‐β3 integrins led to decidual infiltration of NK cells and trophoblast apoptosis, resulting in miscarriage, a process that could be averted through NK‐cell depletion or blockade of NKp46 or FcɣRIIIa receptors.^[^ ^53^ ^]^ Furthermore, inhibition of M1 macrophage polarization has been shown to rescue embryo resorption.^[^ ^54^ ^]^ Except for the targeting capacity of CD16Ab, GC‐Exo‐CD16Ab modulated immune homeostasis via the contribution of MSC‐Exos and GC, involving IL‐17 and TNF signaling pathway, cytokine‐cytokine receptor interaction, chemokine signaling pathway, phagosome, and complement and coagulation cascades. The released GCs regulate biological processes via binding with the GC receptors. Within the cell, GC acts in three ways.^[^ ^55^ ^]^ First, the cortisol‐GC receptor complex moves into the nucleus, where it binds as a homodimer to DNA sequences called GC‐responsive elements. The resulting complex recruits either coactivator or corepressor proteins that modify the structure of chromatin, thereby facilitating or inhibiting the assembly of the basal transcription machinery and the initiation of transcription by RNA polymerase II. Second, regulation of other GC‐responsive genes involves interactions between the cortisol‐GC receptor complex and other transcription factors, such as nuclear factor‐kB. The third mechanism involves the nongenomic pathways. For example, GCs induce the production of reactive oxygen species/reactive nitrogen species and DNA damage through an iNOS‐mediated pathway in breast cancer.^[^ ^56^ ^]^ As for MSC‐Exos, it has been reported that thrombospondin 1 on Exos activates TGFβ, and inhibits the proliferation, activation, and cytotoxicity of NK cells through the TGFβ/Smad2/3 signaling.^[^ ^43^ ^]^ Additionally, MSC‐EXOs were found to modulate macrophage phenotype to regulate inflammatory microenvironment. MSC‐EXOs have been reported to deliver miR‐148a to target Kruppel‐like factor 6 to promote anti‐inflammatory macrophages by inhibiting the STAT3 pathway.^[^ ^57^ ^]^ MSC‐Exos deliver miR‐125a‐5p to increase M2 macrophage polarization, promote angiogenesis, and attenuate fibroblast proliferation and activation via targeting Klf13, Tgfbr1, and Daam1.^[^ ^58^ ^]^

This study highlights the dysregulated immune environment in early miscarriage, characterized by the accumulation and activation of CD16^+^ NK cells and CD16^+^ macrophages in the decidua. To address this challenge, we developed a novel drug delivery platform, GC‐Exo‐CD16Ab, capable of precisely targeting decidual immune cells. By encapsulating GCs in MSC‐Exos and conjugating them with CD16Ab, we achieved enhanced tissue‐specific delivery and controlled release of GCs. GC‐Exo‐CD16Ab demonstrated impressive therapeutic efficacy in a mouse model of LPS‐induced abortion. It significantly improved pregnancy outcomes, restored placental and fetal morphology, and inhibited decidual cell apoptosis. Importantly, GC‐Exo‐CD16Ab effectively suppressed the activation of toxic CD11b^+^ NK cell subsets and pro‐inflammatory IRF5^+^ M1 macrophages in the LPS‐treated mice, emphasizing its ability to modulate the immune microenvironment. These findings underscore the potential of GC‐Exo‐CD16Ab as a promising therapeutic strategy for the treatment of miscarriage and other pregnancy‐related diseases facing immune dysfunction, due to the anti‐inflammatory property of GC, the immune cells‐targeting ability of CD16Ab, and the accumulation of MSC‐Exos in uterus. In addition, the Exos are engineerable. The drug loaded in the Exos or surface antibody modification can be adjusted for specific diseases.

However, it is important to acknowledge the limitations of this study. First, exploring the trajectory and transport mechanism of GC‐Exo‐CD16Ab at the maternal‐fetal interface can help us understand its pharmacokinetics and provide valuable insights for future research endeavors. Second, although the weight and morphology of organs, as well as the parameters of routine blood test and blood biochemistry assay, show similar outcomes between the engineered Exos and the control group, more studies should be performed to figure out the potential side effects of the engineered Exos on maternal health, placental function, and fetal development. Moreover, the in vivo safety assessment was tested at Gd13.5. Long‐term effects of Exos on maternal and fetal health, even on the health of the offspring, are important to evaluate the biosafety of the engineered Exos, especially for clinical translation. In addition, although we have demonstrated significant impacts of GC‐Exo‐CD16Ab on the uterus, we cannot disregard its potential regulatory effects on other organs. Therefore, further investigation into the contribution of natural or engineered Exos to different organs is warranted.

Translating engineered Exos from preclinical studies to clinical applications involves several potential challenges and considerations that need to be addressed. First, physiological differences between mice and humans need to be considered, such as metabolic rates, drug clearance speeds, placental structure, gestational length, and the development of pregnancy‐related disorders. Therefore, before clinical translation, rigorous preclinical studies regarding drug dosing regimens, drug metabolism studies, safety assessments, and efficacy, need to be conducted in large animal models or even humans.^[^ ^59^ ^]^ Second, developing scalable and cost‐effective manufacturing processes for producing engineered Exos is essential for their clinical translation. Quality control measures should be implemented to ensure the consistency of engineered Exos.^[^ ^60^ ^]^ Third, meeting regulatory requirements and obtaining approval from regulatory agencies for conducting clinical trials with engineered Exos is a critical step in the translation to clinical applications. Compliance with Good Manufacturing Practice guidelines is necessary for ensuring the quality and safety of engineered Exos. Finally, designing well‐controlled clinical trials to evaluate the safety and efficacy of engineered Exos in specific patient populations is essential. Considerations such as patient selection criteria and outcome measures should be carefully planned to generate meaningful data.

## 4 Experimental Section

### Inclusion and Analysis of Human Samples

The study was approved by the Ethics Committee of Shenzhen Zhongshan Obstetrics & Gynecology Hospital (SZOGH, formerly Shenzhen Zhongshan Urology Hospital, China) with Ethical Approval Number SZZSECHU‐202012, and written informed consent was obtained from each participant. Fresh decidua and peripheral blood samples were collected from patients at the Department of Obstetrics and Gynecology of SZOGH. The decidual samples from the NP group were obtained by elective pregnancy terminations. Analyses of CD14^+^CD16^+^ and CD14^+^CD16^−^ cells were performed using decidual samples from 14 NP controls and 7 females with first‐trimester SM. Peripheral blood samples from 15 patients were pooled per experiment to evaluate the in vitro function of GC‐Exo‐CD16Ab. Summarily, peripheral blood samples from 420 females were used in this part.

For quantitative real‐time PCR analysis (qRT‐PCR), decidual samples from 21 females with SM, including 3 recurrent miscarriage patients and 18 females who experienced only one miscarriage, and 18 first‐trimester NP controls were retrieved from the tissue bank of SZOGH. For double immunohistochemistry (IHC) analysis, 20 endometrial samples, 13 SM, and 26 NP decidual samples during the first trimester were included from the same tissue bank. Patients with endocrine or metabolic diseases, uterine abnormalities, infections, and fetal chromosome abnormalities were excluded from the whole study. The baseline characteristics of the patients are described in Table S1 (Supporting Information).

Fresh decidual tissues were cut and digested into single cell suspensions to analyze the phenotype, intracellular cytokines, and the phagocytic function of macrophages. Experimental details are shown in the Supporting Information. The detailed methods of qRT‐PCR analysis and IHC staining of human samples are also shown in the Supporting Information.

### Synthesis of GC‐Exo‐CD16Ab

The purification of MSC‐Exos and the preparation and characterization of GC‐Exo‐CD16Ab are shown in the Supporting Information.

### Uptake of Exos by NK Cells and Macrophages

*T*o evaluate the uptake efficiency of Exos by NK cells and macrophages, human peripheral NK cells and induced macrophages stained with PKH67 (Applygen, C0023, China) were seeded into 96‐well plates (5 × 10^4^ cells/well), and then incubated with PBS/Exo (20 µg mL^−1^) stained with PKH26 (Applygen, C0025, China) for 12 h. Cells were analyzed using a High Content Analysis System (PerkinElmer, USA).

The purification and culture of NK cells and macrophages are shown in the Supporting Information. The methods used to evaluate the effect of GC‐Exo‐CD16Ab on NK cells and macrophages in vitro are also shown in the Supporting Information.

### Mouse Abortion Model and Treatment

All animal operations are approved by the Animal Ethics Committee of Renmin Hospital of Wuhan University (Ethical Approval No. 20201207). Eight‐week‐old female C57BL/6 mice were randomly assigned to experimental groups. Ten‐week‐old male BALB/c mice were used for mating. Gestational day 0.5 (Gd0.5) was determined by observation of the copulation plugs using a sterile vaginal probe. Pregnant females were intraperitoneally injected with 0.25 mg kg^−1^ LPS at Gd 7.5 to induce abortion.^[^ ^41^ ^]^ Detailed information is shown in the Supporting Information.

### Tracking Exos In Vivo

Intravital imaging was used to confirm the dynamic trajectory of Exos in mouse uterus with mouse survival as indicated by blood moving. The distribution of Exos in different organs was further evaluated by an IVIS imaging system. IHC and flow cytometry were used to analyze the targeting capacity of GC‐Exo‐CD16Ab to NK cells and macrophages in vivo. Detailed information is shown in the Supporting Information.

### In Vivo Safety Assessment

The in vivo safety of Exos was verified by H&E staining of organ tissues, organ weight, routine blood test, and blood biochemistry assay.

Detailed methods used to evaluate pregnancy outcomes and decidual immune microenvironment in vivo are shown in the Supporting Information.

Statistical analysis is shown in the Supporting Information.

## Conflict of Interest

The authors declare no conflict of interest.

## Author Contributions

L.W., Z.Y., and Y.S. contributed equally to this work. L.W., Z.Y., and Y.S. conceived and designed the experiments, conducted the study, performed all experimental aspects of the project, analyzed and interpreted data, and prepared the manuscript. G.F., F.D., D.Y., Z.D., J.Y., and R.C. assisted in the experimental aspects of the project. L.Y., X.C., Q.S., and C.H. produced, characterized, and provided engineered and naïve exosomes, and edited the manuscript. Y.C., H.D., L.D., L.L., and T.Y. conceived the idea, provided funds and other resources, and assisted with data interpretation and preparation of the manuscript.

## Supporting information

Supporting Information

## Data Availability

The data that support the findings of this study are available in the supplementary material of this article.
