# Supplementary material for: Targeting Decidual CD16+ Immune Cells with Exosome‐Based Glucocorticoid Nanoparticles for Miscarriage
Source: Adv Sci (Weinh). 2024 Aug 13;12(24):2406370. doi: 10.1002/advs.202406370 (PMC12199590; doi:10.1002/advs.202406370)
Supplement: Supplementary file 1 — Supporting Information [file ADVS-12-2406370-s001.docx]

**Targeting Decidual CD16^+^ Immune Cells with Exosome-Based Glucocorticoid Nanoparticles for Miscarriage**

**Experimental details**

*Processing and analyses of human decidual CD14^+^CD16^+^ and CD14^+^CD16^-^ cells:* Decidual tissues (4–6 g) were cut and digested in RPMI-1640 medium (HyClone, SH30809.0.1, USA) supplemented with type IV collagenase (1.0 mg/mL; Sigma-Aldrich, C4-28, USA) and 1% fetal bovine serum (FBS; Corning, 35-081-cv, USA) for 30 min at 37°C with gentle agitation.^[1]^ The suspension was filtered through 100- and 40-μm meshes, and incubated in Erythrocyte lysis buffer (Absin, abs9241, China) for 20 min at 4°C to decrease erythrocyte contamination, and washed with PBS.

For macrophage phenotype analysis, 10^6^ cells were resuspended in 100 μL PBS with 5 μL Fc receptor blocking solution (BioLegend, 422302, USA) for 10 min, and incubated with appropriate antibodies (Abs) for 15 min at room temperature for cell surface staining. The following reagents were used: FITC anti-human CD45 Ab (BioLegend, 304006, USA), PE-CY5 anti-human CD14 Ab (BioLegend, 301864, USA), PE-CY7 anti-human CD16 Ab (BD Biosciences, 557744, USA), BV785 anti-human CD86 Ab (BioLegend, 305442, USA), PE anti-human CD163 Ab (BioLegend, 333606, USA) and Fix viability dye (Thermo Fisher Scientific, 65-0866-18, USA).

For intracellular cytokine detection, decidual cells were seeded into 12-well plates at a density of 10^6^ cells/mL, and simultaneously treated with Brefeldin A (BioLegend, 420601, USA) and 100 ng/mL lipopolysaccharide (LPS; Sigma-Aldrich, L4391, USA) at 37 °C in 5% CO_2_ for 4 h.^[2]^ After stimulation, cells were collected, washed with PBS, and resuspended in Fc receptor blocking solution. Cell surface staining was performed using PE-CY7 anti-human CD45 Ab (BioLegend, 304016, USA), PE-CY5 anti-human CD14 Ab, BV650 anti-human CD16 Ab (BioLegend, 302042, USA), and Fix viability dye. After washing with PBS, cells were fixed and permeabilized according to the manufacturer’s protocol (BD FACS™ Permeabilizing Solution 2, BD Biosciences, 340973, USA). Permeabilized cells were stained with intracellular cytokine Abs, including FITC anti-human IFN-γ Ab (BioLegend, 502506, USA), APC anti-human TNF-α Ab (BioLegend, 502912, USA), and BV421 anti-human TGF-β Ab (BioLegend, 349613, USA) for 30 min at room temperature.

FITC-dextran (molecular mass 40 kDa, Sigma-Aldrich, FD40S, USA) was used to determine the phagocytic function of macrophages. Decidual cells were seeded into 12-well plates at a density of 10^6^ cells/mL for 3 h. The above cells were then incubated with 0.5 mg/L FITC-dextran at 37°C or at 4°C for 45 min to detect nonspecific binding.^[3]^ After the cells were washed and collected, the percentage of dextran-positive cells was determined by flow cytometry (FCM).

FCM analysis was performed on a Beckman-Coulter DxFlex Analyzer (Beckman, USA). Data were analyzed with CytExpert software 2.4 (Beckman, USA).

*RNA isolation and quantitative real-time PCR analysis:* Total RNAs were isolated from tissues using Qiagen RNeasy Plus Mini Kit (Qiagen, 74134, Germany). Then, reverse transcription was carried out by q*RT-PCR* kit (PrimeScript RT Reagent Kit, Takara, RR047A, Japan; Luna Universal qPCR Master Mix, NEB, M3003L, USA). The primers for genes and the reference gene are listed in Table S2 (Supporting Information). Additionally, data were shown as relative expression compared with the expression of actin, and each sample was repeated at least 3 times.

*IHC staining of human samples:* IHC staining was performed in an automatic immunostaining machine (Leica Bond Rx system, Wetzlar, Germany) using a Bond polymer refine detection kit (Leica Microsystems, Wetzlar, DS9800 and DS9390, Germany). Dual IHC staining for human decidual CD16^+^ cells was performed on formalin-fixed, paraffin-embedded (FFPE) sections using Bond polymer refine DAB and Red detection kits. The image acquisition was completed by the multi-spectral panoramic organization program analysis system on the Olympus VS200 Slide Scanner (Olympus, Tokyo, Japan), and analyzed by the HALO image analysis platform (Indica Labs, NM, USA). The primary antibodies are outlined in Table S3 (Supporting Information).

*Purification of MSC-Exos:* Human umbilical cord mesenchymal stem cells (MSCs) were cultured in Dulbecco’s modified Eagle’s medium/nutrient mixture F-12 Ham (DMEM/F-12, HyClone, SH30023.01, USA) supplemented with 10% FBS in a humidified atmosphere of 95% air and 5% CO_2_ at 37°C. When achieving 75% healing rates, the cell culture supernatant was then replaced with an Exo-free medium. After 24 h, the cell culture supernatant was collected to purify Exos using a TransExo cell media exosome kit (TransGen Biotech, FE401, China) under the guidance of instructions. The obtained suspension of MSC-Exos was stored at −80°C for later use.

*Preparation of GC-Exo-CD16Ab:* Anti-CD16 antibodies used for MSC-Exo modification were assessed through NK cytotoxicity assay. Human peripheral blood NK cells were purified using a human NK Cell Isolation Kit (Miltenyi Biotec, 130-092-657, Germany) at a density of 10^6^ cells/mL and treated with 10 μg/mL anti-CD16 antibodies (Ab1, Sino Biological, 10389-MM41, China; Ab2, ProteinTech, 16559-1-AP, China; Ab3, ProteinTech, 66779-1-Ig, China) for 48 h.^[4]^ The cytotoxicity of NK cells treated with 10 ng/mL IL-15 (Sino Biological, 10360-HNCE, China) was served as a positive control.^[5]^ NK cells were then washed and cocultured with K562 cells at a 10:1 or 5:1 E:T ratio for 4 h. K562 cells were prestained with DiO (Thermo Fisher Scientific, D275, USA). After treatment for another 4 h, 10 μL Propidium Iodide (Sigma-Aldrich, P4170, USA) was added into the coculture system and samples were harvested for FCM. Anti-CD16 (Ab2) at different concentrations (0, 5, 25, 50, 250, 1000, 5000, 10000 μg/mL) was selected for further evaluation and ultimately used to decorate MSC-Exos.

Methylprednisolone sodium succinate (KingYork, H20103047, China), the most used glucocorticoid (GC) in pregnant patients, was used in this study. Exo-GC was prepared by ultrasonic incubation of MSC-Exo (500 μg/mL) and GC (250, 500, 1000 μg/mL). The mixture was sonicated using a Model 505 Sonic Dismembrator with a 0.25-inch tip and the following settings: 20% amplitude, 6 cycles of 30 s on/off for 3 min with a 2 min cooldown between each cycle.^[6]^ Subsequently, the mixture was incubated at 37 °C for 1 h to recover the Exo membranes. Excess free GCs were removed by washing five times using 100-KDa ultrafiltration tubes. The conjugation of CD16Ab to the surface of Exo-GC was developed using click reaction.^[7]^ Briefly, Exo-GC (10^13^ particles) in 10 mL PBS was mixed with 4 μM DBCO-sulfo-NHS ester (Sigma-Aldrich, 762040, USA) on a rotator for 6 h at room temperature. CD16Ab (Ab2, 2 μM) was incubated with 20 μM azide-sulfo-NHS (Yusiyy, China) on the rotator for 6 h at room temperature. After washing with PBS, Exo-GC with DBCO group was mixed with azide-CD16Ab at 4 °C for 24 h to form GC-Exo-CD16Ab. Excess free azide-CD16Abs were removed by washing five times using 300-KDa ultrafiltration tubes.

*Characterization of GC-Exo-CD16Ab:* GC-Exo-CD16Ab was characterized by transmission electron microscopy, nanoparticle tracking analysis (NTA, NanoSight NS300, UK), western blotting using an automated Wes Capillary System (ProteinSimple, USA), and hemolytic property. Primary antibodies used for western blotting were calnexin, ALIX, and CD63 at a 1/25 dilution (Abcam, ab10286, ab275377, ab271286, UK).

The amount of GC loaded in GC-Exo-CD16Ab was determined using a high-performance liquid chromatography system (Agilent 1260 Autosampler, Agilent Technologies, USA) coupled with electrospray tandem mass spectrometry (API 4000 Triple Quadrupole system, SCIEX, USA). For in vitro GC release profiles, free GC and GC-Exo-CD16Ab (200 μg/mL GC) were placed in dialysis bags (MWCO 3500 Da), which were placed in PBS containing polysorbate 80 (0.5%, w/v) at 37 °C with gentle shaking (100 rpm). Subsequently, GC diffused from the dialysis bags into the PBS solution. At predetermined time points, samples were drawn from the PBS solution and replaced with an equal volume of prewarmed fresh medium. Finally, the samples were analyzed with a microplate reader (BioTek Synergy H1 Plate Reader, Agilent, USA).

Anti-CD16 antibody decorated on MSC-Exos was quantified according to the standard curve. Resuspended Exos (30 μL) were lysed with an equal volume of radioimmunoprecipitation (RIPA) lysis buffer. The lysates were centrifuged at 12000 g at 4°C for 15 min to collect the supernatant. The above samples and anti-CD16 antibodies at different concentrations (0, 0.5, 1, 2, 4, 8, 16, 32, 64 mg/L) were measured using an automated Wes Capillary System. NTA was used to quantify the number of Exos. The antibody loading efficiency was calculated using the following equation. Antibody loading efficiency (μg/10^10^ particles) = weight of antibody/number of engineered Exos.

*Cell culture:* Human peripheral blood NK cells were purified using a human NK Cell Isolation Kit. NK cells were cultured in RPMI-1640 medium, supplemented with 10% FBS and 1% penicillin/streptomycin/amphotericin B (Beyotime, C0224, China) at 37°C in a humidified air of 5% CO_2_. Human peripheral monocytes were purified using human CD14 MicroBeads (Miltenyi Biotec, 130-050-201, Germany). The separated monocytes were then stimulated with 5 ng/mL rhGM-CSF (Beyotime, P5286, China) in RPMI-1640 medium supplemented with 10% FBS and 1% penicillin/streptomycin/amphotericin B for 6 days to induce M1 macrophages. The monocytes were stimulated with 50 ng/mL rhM-CSF (Beyotime, P5313, China) for 6 days to induce M2 macrophages.

*The effect of GC-Exo-CD16Ab on NK cells:* NK cells were seeded in 12-well plates (5 × 10^5^ cells/well), and treated with PBS/GC/Exo-GC/Exo-CD16Ab/GC-Exo-CD16Ab (20 μg/mL Exos containing 10^-6^ M GC) for 48 h. In order to evaluate the NK activation receptors, NK cells were then washed with PBS, and incubated with Pacific Blue anti-human CD45 Ab (BioLegend, 368540, USA), BV785 anti-human CD3 Ab (BioLegend, 344842, USA), BV605 anti-human CD56 Ab (BioLegend, 362538, USA), PE anti-human NKp30 Ab (Invitrogen, 12-3379-42, USA), APC anti-human NKp46 Ab (BioLegend, 331917, USA), and FITC anti-human NKG2D Ab (BioLegend, 320819, USA) for 15 min. For NK inhibitory receptors detection, cells were incubated with Pacific Blue anti-human CD45 Ab, BV785 anti-human CD3 Ab, BV605 anti-human CD56 Ab, FITC anti-human CD158a Ab (BD Biosciences, 556062, USA), PE anti-human CD158b Ab (BD Biosciences, 559785, USA), and APC anti-human NKG2A Ab (BioLegend, 375107, USA) for 15 min. To analyze cytotoxic granules, cell surface staining was performed using Pacific Blue anti-human CD45 Ab, BV785 anti-human CD3 Ab, BV605 anti-human CD56 Ab, and Fix viability dye. After washing with PBS, cells were fixed, permeabilized, and stained with Alexa fluor 647 anti-human perforin Ab (BD Biosciences, 563576, USA), Alexa fluor 488 anti-human granulysin Ab (BD Biosciences, 558254, USA), and PE anti-human granzyme B Ab (BD Biosciences, 561142, USA) for 30 min at room temperature. Cells were then washed and analyzed by FCM. After treatment with PBS/GC/Exo-GC/Exo-CD16Ab/GC-Exo-CD16Ab for 48 h, NK cells were washed and cocultured with K562 cells at a 12.5:1 E:T ratio for 4 h to assess NK cytotoxicity by FCM.

*The effect of GC-Exo-CD16Ab on macrophages:* To evaluate the effect of GC-Exo-CD16Ab on macrophage migration, macrophages were seeded in 96-well plates (6 × 10^3^ cells/well), and treated with PBS/GC/Exo-GC/Exo-CD16Ab/GC-Exo-CD16Ab (20 μg/mL Exos containing 10^-6^ M GC) for 48 h. The trajectory of macrophages was analyzed by a High Content Analysis System.

For analyses of macrophage phenotype and phagocytic activity, cells were seeded in 12-well plates (5 × 10^5^ cells/well), and treated with PBS/GC/Exo-GC/Exo-CD16Ab/GC-Exo-CD16Ab (20 μg/mL Exos containing 10^-6^ M GC) for 48 h. Cells were then washed and incubated with appropriate Abs or FITC-dextran. Please refer to *Processing and analyses of human decidual CD14^+^CD16^+^ and CD14^+^CD16^-^ cells* (Supporting Information) for detailed methods.

*CCK8 assay:* To evaluate cell proliferation, NK cells and macrophages were seeded in 96-well plates (6 × 10^3^ cells/well) and incubated with PBS/GC/Exo-GC/Exo-CD16Ab/GC-Exo-CD16Ab (20 μg/mL Exos containing 10^-6^ M GC) for 0 to 72 h. CCK8 solution (Sangon Biotech, E606335, China) was added into the culture medium and further cultured for 4 h at 37°C. Finally, the absorbance at 450 nm was measured with a microplate reader.

*Cytometric bead array:* Cytokines in cell culture supernatant were measured with the LEGENDplex Human CD8/NK Panel or Human Macrophage/Microglia Panel (BioLegend, USA) according to the manufacturer’s instructions.

*Mouse abortion model and treatment:* In order to evaluate the effects of GC-Exo-CD16Ab and TGFβ-Exo-CD16Ab on mouse pregnancy outcomes, mice were divided into 6 subgroups (n = 4–6/group): (1) Naive-ctrl group, (2) LPS-model group, (3, 4) GC-Exo-CD16Ab/TGFβ-Exo-CD16Ab prevention group [injected with 10^10^ Exos every 2 days at Gd2.5, Gd4.5, Gd6.5 (i.v.), and then injected with LPS at Gd7.5 (i.p.) ], and (5, 6) GC-Exo-CD16Ab/TGFβ-Exo-CD16Ab treatment group [injected with LPS at Gd7.5 (i.p.) and 10^10^ Exos every 2 days at Gd7.5, Gd9.5, and Gd11.5 (i.v.) ]. For evaluating the effects of various structures of GC-Exo-CD16Ab on mouse pregnancy outcomes, mice were divided into 6 subgroups (n = 3–6/group): (1) Naive-ctrl group, (2) LPS-model group, (3) GC treatment group, (4) Exo-GC treatment group, (5) Exo-CD16Ab treatment group, and (6) GC-Exo-CD16Ab treatment group. Except for the naïve-ctrl group injected with saline, the other groups were all intraperitoneally injected with 0.25 mg/kg LPS at Gd 7.5. All the groups were intravenously injected with PBS/GC/Exo-GC/Exo-CD16Ab/GC-Exo-CD16Ab every 2 days on Gd7.5, Gd9.5, and Gd11.5. All mice were sacrificed at Gd13.5 to examine pregnancy outcomes. Peripheral blood, organs (heart, liver, spleen, lungs, kidneys, and ovaries), as well as placental tissues including decidual tissues and fetus were collected for the following experiments.

*Tracking Exos in vivo:* Intravital imaging was used to confirm the dynamic trajectory of Exos in mouse uterus with mouse survival as indicated by blood moving. Pregnant female mice were anesthetized, and injected with AIE dye-labeled Exos and FITC-dextran (molecular mass 70 kDa, Sigma-Aldrich, 90718, USA) to image blood vessels. A midline incision was then made in the abdominal muscle and fetuses were removed from the abdominal cavity to minimize motion from the dam’s breathing. The decidua basalis side of the uterus was covered with a circular window via a vacuum. The window with a 5-mm inner diameter and 1.5-mm depth was covered with a glass coverslip. Two-photon microscopy (Leica, Germany) was then used to image Exos.^[8]^

To further evaluate the distribution of Exos in vivo, pregnancy C57BL/6 mice (3 mice/group) were intravenously injected with PBS/Exo/GC-Exo-CD16Ab (10^10^ particles/100 μL). Exos were pre-dyed with AIE dye.^[9]^ At selected time points, mice were sacrificed. Organs including the heart, liver, spleen, lungs, kidneys, and uterus were isolated and imaged via an IVIS imaging system (PerkinElmer, USA). The fluorescence signals in organs were quantified as radiant efficiency. To analyze the targeting capacity of GC-Exo-CD16Ab to NK cells and macrophages in vivo, mouse uteruses were then fixed in 30% sucrose and dehydrated in 4% polyformaldehyde solution to avoid the influence of ethanol and xylene on fluorescent dye used for Exo labeling.^[10]^ Dehydrated tissue was frozen with OCT compound. Frozen sections were dyed with immune cell markers and a multiplex IHC kit (Panovue, China). The primary antibodies are outlined in Table S3 (Supporting Information). Flow cytometry was used to confirm the targeting capacity of GC-Exo-CD16Ab to decidual immune cells (6 mice/group). Decidual tissues were cut and digested in Hanks medium supplemented with collagenase (1.0 mg/mL; Sigma-Aldrich, C2139, USA), hyaluronidase (0.28 mg/mL; Sigma-Aldrich, H3506, USA) and BSA (1.0 mg/mL; Sigma-Aldrich, A7906, USA) for 30 min at 37°C with gentle agitation. The suspension was filtered through 100- and 40-μm meshes, incubated in Erythrocyte lysis buffer (Absin, abs9241, China) for 8 min at 4°C to decrease erythrocyte contamination, and washed with PBS. Cells were incubated with anti-mouse Fc receptor blocking solution for 10 min, and then incubated with appropriate Abs for 15 min at room temperature for cell surface staining. The following reagents were used: FITC anti-mouse CD45 Ab (BioLegend, 157214, USA), PE anti-mouse NK1.1 Ab (BioLegend, 156504, USA), APC anti-mouse F4/80 Ab (BioLegend, 123116, USA), BV605 anti-mouse CD11b Ab (BioLegend, 101257, USA) and Fix viability dye (Thermo Fisher Scientific, 65-0866-18, USA).

In order to evaluate the GC delivery capacity of the Exo-GC system, GCs connected with Cy5.5 (Yusiyy, China) were used to construct the engineered Exos. PBS, GC or Exo-GC was then injected into the pregnancy mice at Gd7.5, and imaged via an IVIS imaging system.

*Evaluation of pregnancy outcomes:* Mice were sacrificed at Gd13.5 to examine fetal weight. Characterization of placental and fetal phenotypes included 1–3 randomly selected conceptuses for each dam. H&E staining was used to assess placental morphology and fetal growth. Area, diameter, and thickness of the whole utero-placental unit/labyrinth (Lab), junctional zone (Jz), mesometrial decidua (basalis, Db), and mesometrial lymphoid aggregate of pregnancy (MLAp) were measured. Fetal length was also detected.

*IHC staining of mouse samples:* Single IHC staining for mouse CD16 protein was performed on FFPE sections using a Bond polymer refine DAB detection kit. Multiplex IHC staining for the analysis of mouse decidual immune microenvironment was performed on FFPE sections using a PANO IHC kit (Panovue, China). The primary antibodies are outlined in Table S3 (Supporting Information).

*Statistical Analysis:* The single-cell RNA sequencing data of decidual immune cells were downloaded from the GSA database (https://ngdc.cncb.ac.cn/gsa/) with accession number CRA002181. The data contains a total of 24 decidual tissues. Raw data were processed using Cell Ranger (version 3.0.2) and aligned to the hg19 genome reference to generate the gene expression matrix. Downstream analyses were performed using the Seurat (version 4.3.0) package in R (version 4.3.1). Low-quality cells were removed based on the criteria of nFeature_RNA > 500 & nFeature_RNA < 3000 & percent.mt < 10%. The doublet cells were removed by the DoubletFinder (version 2.0.3) package. Cell annotation and visualization were carried out after removing batch effects using the canonical correlation analysis (CCA) algorithm. Cells were then categorized into different groups based on the expression of target genes. Differentially expressed genes (DEGs) between CD16^+^ and CD16^-^ immune cells were identified using the FindAllMarkers function in Seurat (version 4.3.0). The expression of DEGs in different groups was visualized by heatmap. To explore the possible functions and signaling pathways mediated by DEGs, Gene ontology (GO) and Kyoto Encyclopedia of Genes and Genomes (KEGG) analyses were performed on DEGs using the clusterProfiler (version 4.8.1) package. Unless otherwise noted, the above analyses all used default parameters.

mRNA sequencing of the placenta of mice was performed by Personal Biotechnology Company (Shanghai, China). DEGs were identified using Deseq2 (|log2FoldChange| > 1, P-value < 0.05). The expression of DEGs, and GO and KEGG analyses were analyzed using https://www.bioinformatics.com.cn/.

Other statistical analysis was performed using Prism software (GraphPad, version 9.4.1). Differences among three or more groups with only one variable were assessed using one-way ANOVA with the Tukey post hoc test. Differences between only two groups with one variable were assessed using an unpaired two-tailed t-test. Two-way ANOVA with the Tukey post hoc test was used for comparison of two or more groups with two variables. Data are shown as mean ± Standard Error of the Mean (SEM) or mean ± Standard Deviation (SD) according to different experiments. Significant differences are indicated by *p < 0.05, **p < 0.01, ***p < 0.001, ****p < 0.0001, and no significant differences are indicated by “n.s.”. Letters a, b, and c were used to show statistically significant differences in the percentages of absorbed fetuses among groups via the chi-squared test.

**References**

[1] Y. H. Li, W. H. Zhou, Y. Tao, S. C. Wang, Y. L. Jiang, D. Zhang, H. L. Piao, Q. Fu, D. J. Li, M. R. Du, *Cell Mol Immunol* **2016**, *13*, 73.

[2] A. Sinistro, C. Ciaprini, S. Natoli, E. Sussarello, F. C. Carducci, C. Almerighi, M. Capozzi, F. Bolacchi, G. Rocchi, A. Bergamini, *Immunology* **2007**, *122*, 362.

[3] C. Wang, X. Yu, Q. Cao, Y. Wang, G. Zheng, T. K. Tan, H. Zhao, Y. Zhao, Y. Wang, D. Harris, *BMC Immunol* **2013**, *14*, 6.

[4] A. Jewett, A. Teruel, M. Romero, C. Head, N. Cacalano, *Cancer Immunol Immunother* **2008**, *57*, 1053.

[5] Y. H. Choi, E. J. Lim, S. W. Kim, Y. W. Moon, K. S. Park, H. J. An, *J Immunother Cancer* **2019**, *7*, 168.

[6] X. Zheng, K. Sun, Y. Liu, X. Yin, H. Zhu, F. Yu, W. Zhao, *J Control Release* **2023**, *353*, 675.

[7] H. Song, X. Chen, Y. Hao, J. Wang, Q. Xie, X. Wang, *J Nanobiotechnology* **2022**, *20*, 431.

[8] Q. Huang, M. A. Cohen, F. C. Alsina, G. Devlin, A. Garrett, J. McKey, P. Havlik, N. Rakhilin, E. Wang, K. Xiang, P. Mathews, L. Wang, C. Bock, V. Ruthig, Y. Wang, M. Negrete, C. W. Wong, P. K. L. Murthy, S. Zhang, A. R. Daniel, D. G. Kirsch, Y. Kang, B. Capel, A. Asokan, D. L. Silver, R. Jaenisch, X. Shen, *Science* **2020**, *368*, 181.

[9] a) Y. Xu, W. Yang, D. Yao, K. Bian, W. Zeng, K. Liu, D. Wang, B. Zhang, *Chem Sci* **2020**, *11*, 419; b) W. Xu, M. M. S. Lee, Z. Zhang, H. H. Y. Sung, I. D. Williams, R. T. K. Kwok, J. W. Y. Lam, D. Wang, B. Z. Tang, *Chem Sci* **2019**, *10*, 3494.

[10] J. He, Y. Cao, Q. Zhu, X. Wang, G. Cheng, Q. Wang, R. He, H. Lu, Y. Weng, G. Mao, Y. Bao, J. Wang, X. Liu, F. Han, P. Shi, X. Z. Shen, *Immunity* **2024**, *57*, 106.

**Supplementary tables**

**Table S1.** Clinical characteristics of the study population.

|  | Number | Age (y) | BMI (kg/m^2^) | Gestational age (week) |
| --- | --- | --- | --- | --- |
| Population included for the analyses of CD14^+^CD16^+^ and CD14^+^CD16^-^ cells | | | | |
| NP | 14 | 34.43 ± 1.76 | 21.25 ± 0.84 | 6.95 ± 0.28 |
| SM | 7 | 33.29 ± 1.96 | 22.06 ± 0.77 | 7.55 ± 0.51 |
| Population included to evaluate the function of GC-Exo-CD16Ab in vitro | | | | |
| Female patients with reproductive failure | 420 | 34.65 ± 0.21 | 21.21 ± 0.13 | / |
| Population included for quantitative real-time PCR analysis | | | | |
| NP | 18 | 32.61 ± 1.70 | 21.91 ± 0.56 | 7.30 ± 0.22 |
| SM | 21 | 33.81 ± 1.11 | 21.59 ± 0.48 | 7.56 ± 0.33 |
| Population included for double immunohistochemistry analysis | | | | |
| Endo | 20 | 32.10 ± 0.87 | 22.30 ± 0.58 | / |
| NP | 26 | 33.77 ± 1.91 | 21.41 ± 0.76 | 7.43 ± 0.47 |
| SM | 13 | 33.69 ± 0.78 | 21.80 ±0.65 | 7.40 ± 0.19 |

Note: BMI, body mass index; NP, normal pregnancy; SM, spontaneous miscarriage; Endo, healthy endometrium; Data are presented as mean ± SEM. There were no significant differences between groups via Unpaired two-tailed t-test or One-way ANOVA.

**Table S2.** The primers for human immune response-related genes and internal reference gene.

| Gene Symbol | Forward primer (5’-3’) | Reverse primer (5’-3’) |
| --- | --- | --- |
| *NKG2A* | TTGCTGGCCTGTACTTCGA | CCAAACCATTCATTGTCACCC |
| *IL-4* | CCGAGTTGACCGTAACAGACAT | GTCCTTCTCATGGTGGCTGTAG |
| *TGF-β1* | CCCAGCATCTGCAAAGCTC | GTCAATGTACAGCTGCCGCA |
| *TGF-β2* | TGCCGCCCTTCTTCCCCTC | GGAGCACAAGCTGCCCACTGA |
| *CD16* | ATGTGTCTTCAGAGACTGTGAAC | TTTATGGTCCTTCCAGTCTCTTG |
| *CD11b* | CAGCCTTTGACCTTATGTCATGG | CCTGTGCTGTAGTCGCACT |
| *TNF-a* | CCCAGGGACCTCTCTCTAATCA | GCTACAGGCTTGTCACTCGG |
| *IL-1β* | CCAAAGAAGAAGATGGAAAAGCG | GGTGCTGATGTACCAGTTGGG |
| Actin | GCCTTTGCCGATCCGC | GCCGTAGCCGTTGTCG |

**Table S3.** Summary of primary antibodies and immunohistochemical technique.

| Antibody | Source | Product No. | Clone | Dilution | Antigen retrieval |
| --- | --- | --- | --- | --- | --- |
| Mouse |  |  |  |  |  |
| CD16 | Affinity Biosciences | DF7007 | Polyclonal | 1:500 | EDTA (pH 8.95-9.05) |
| MCT1 | ProteinTech | 20139-1-AP | Polyclonal | 1:1000 | EDTA (pH 8.95-9.05) |
| PCNA | Abcam | ab92552 | EPR3821 | 1:250 | EDTA (pH 8.95-9.05) |
| Caspase 3 | CST | 9665S | 8G10 | 1:500 | EDTA (pH 8.95-9.05) |
| CD11b | Abcam | ab133357 | EPR1344 | 1:1000 | EDTA (pH 8.95-9.05) |
| F4/80 | Beyotime | AG4753 | / | 1:500 | EDTA (pH 8.95-9.05) |
| IRF5 | Beyotime | AF2488 | / | 1:200 | EDTA (pH 8.95-9.05) |
| CD206 | CST | 24595S | E6T5J | 1:800 | EDTA (pH 8.95-9.05) |
| GrB | CST | 46890S | D6E9W | 1:200 | EDTA (pH 8.95-9.05) |
| NK1.1 | Thermo Fisher Scientific | MA1-70100 | PK136 | 1:50 | EDTA (pH 8.95-9.05) |
| Human |  |  |  |  |  |
| CD16 | CST | 24326S | D1N9L | 1:100 | EDTA (pH 8.95-9.05) |
| CD56 | Gene Tech | GT200529 | 123C3 | 1:1200 | EDTA (pH 8.95-9.05) |
| CD68 | Novocastra | NCL-L-CD68 | 514H12 | 1:80 | EDTA (pH 8.95-9.05) |
| CD8 | Novocastra | NCL-L-CD8 | 4B11 | 1:300 | Citrate (pH 5.80-6.00) |

Note: EDTA: Ethylenediaminetetraacetic acid.

**Supplementary figures**

**
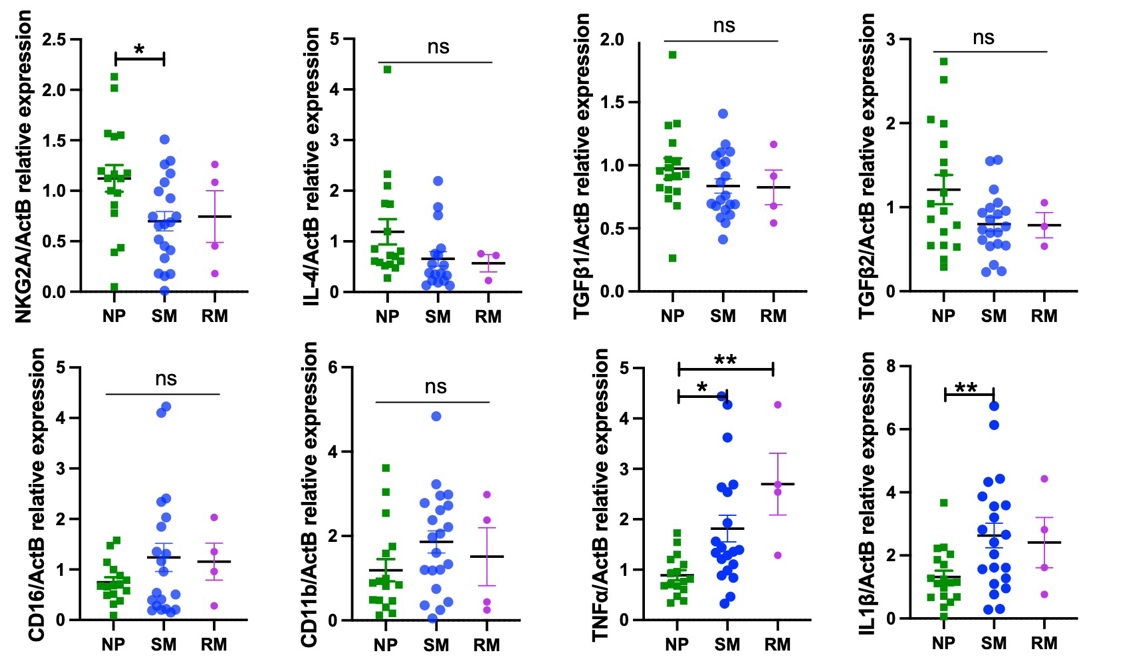
**

**Figure S1.** The expression of immune response-related genes (*NKG2A, IL-4, TGF-β1, TGF-β2, CD16, CD11b, TNF-α, IL-1β*) in decidua of females with NP (*n* = 18), SM (*n* = 21) or RM (*n* = 4). One-way ANOVA was used. All data are represented as mean ± SEM. NP, normal pregnancy; SM, spontaneous miscarriage; RM, recurrent miscarriage. **p* < 0.05, ***p* < 0.01, and no significant differences are indicated by “n.s.”.


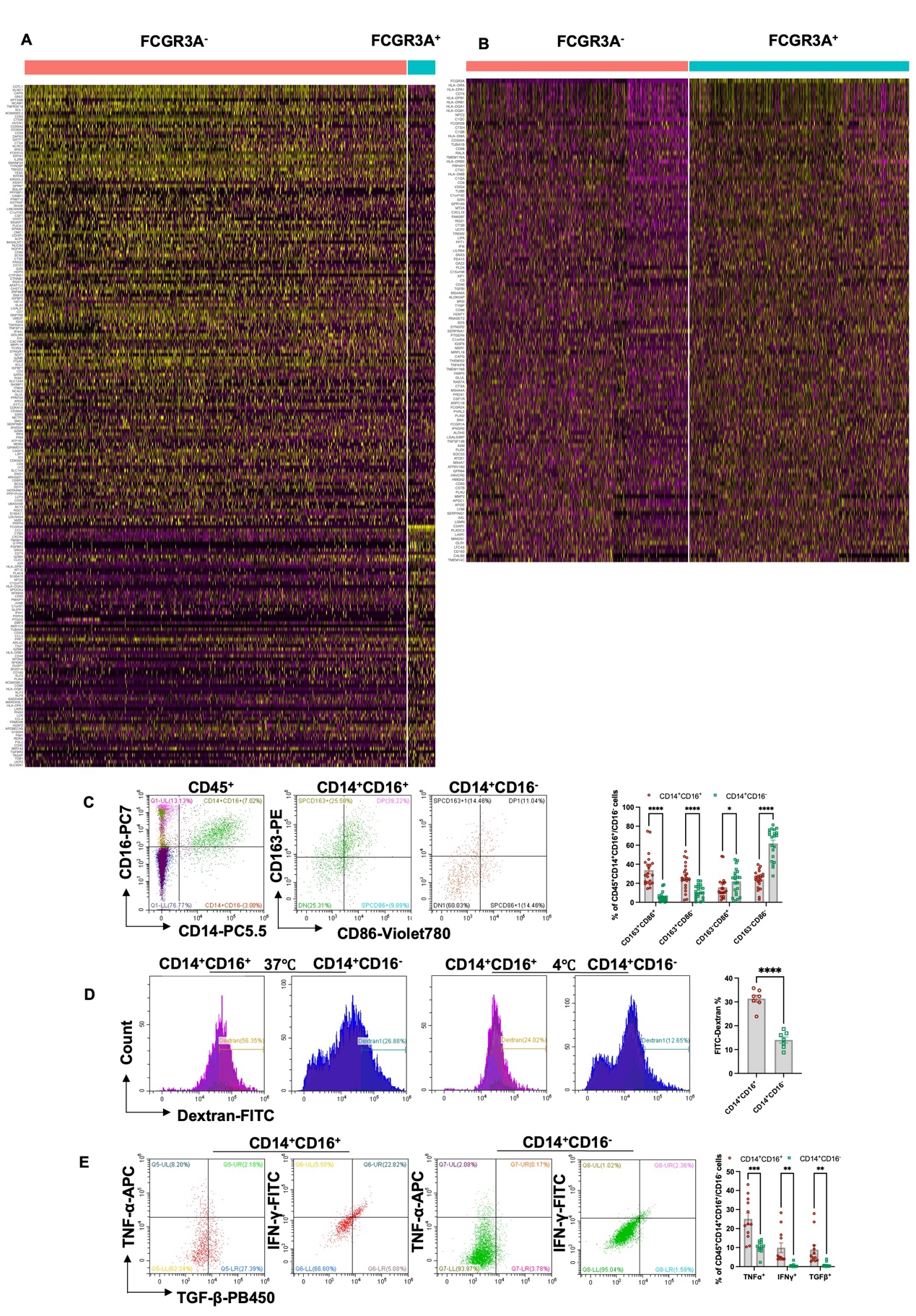


**Figure S2.** The difference between CD16^+^ and CD16^-^ human immune cells. A) Heatmaps of highly expressed genes in CD16^+^ and CD16^-^ human decidual NK cells and B) macrophages (data from the GSA database with accession number CRA002181). C) Representative FCM images and quantitative analysis of the percentages of CD163^+^ and CD86^+^ cells (C, *n* = 21 per group), D) Dextran^+^ cells (D, *n* = 7 per group), and E) TNF-α^+^, IFN-γ^+^ and TGF-β^+^ cells (E, *n* = 12 per group) in decidual CD14^+^CD16^+^ and CD14^+^CD16^-^ cells. Unpaired two-tailed t-tests (C–E) were used. All data are represented as mean ± SEM. *p < 0.05, ***p* < 0.01, ****p* < 0.001, *****p* < 0.0001, and no significant differences are indicated by “n.s.”.


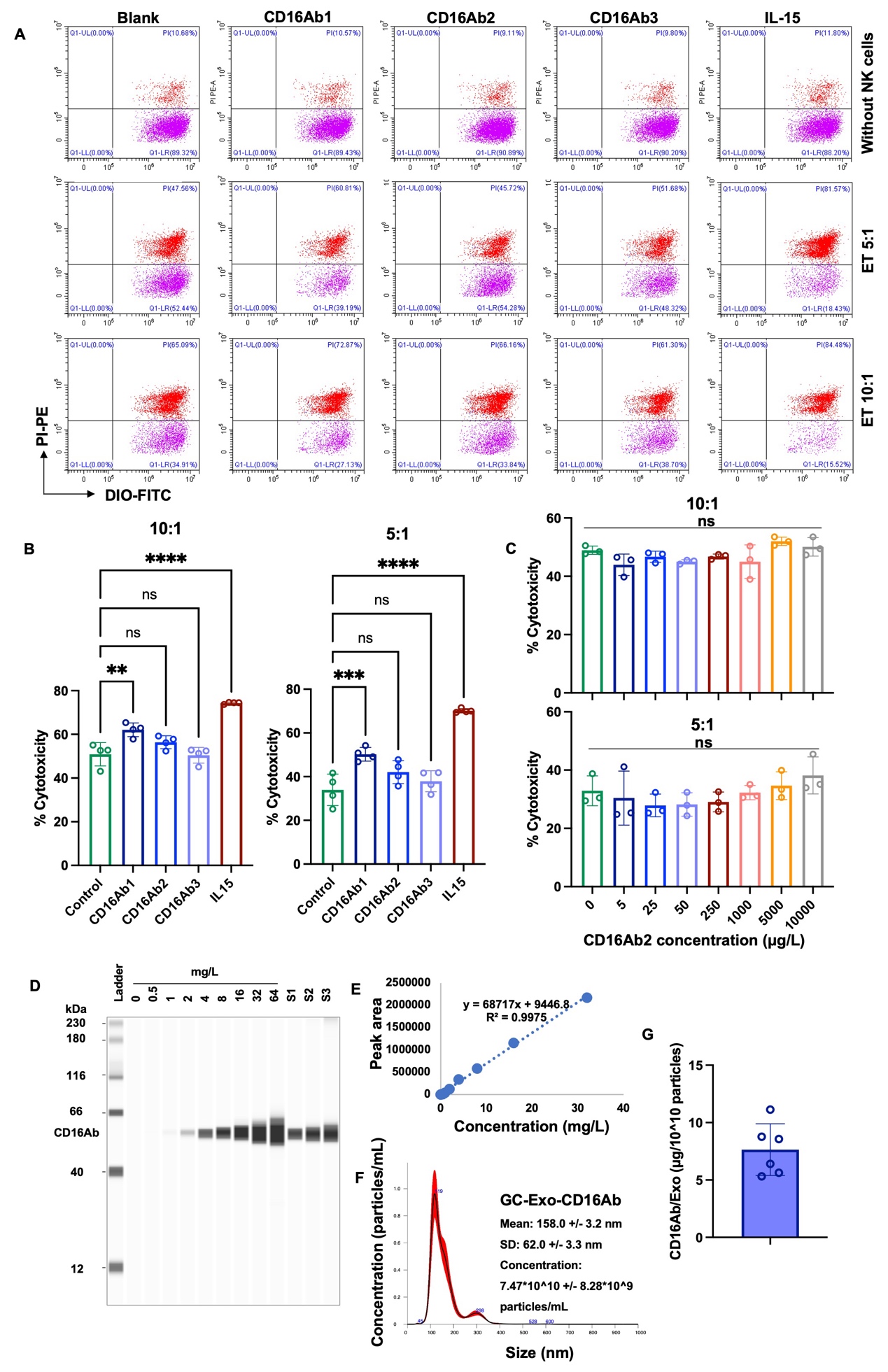


**Figure S3.** The selection and decoration of CD16Ab on Exos. A) Representative FCM images and B) quantitative analysis of the cytotoxicity of NK cells pre-treated with different CD16Abs. IL-15 served as a positive control. ET at 10:1 and 5:1. *n* = 4 independent experiments. C) The NK cytotoxicity of CD16Ab2 at various concentrations. ET at 10:1 and 5:1. *n* = 3 independent experiments. D–G) Decoration and quantitation of CD16Ab2 on engineering exosomes. *n* = 6 samples. One-way ANOVA was used when compared with the control groups. All data are represented as mean ± SD. ***p* < 0.01, ****p* < 0.001, *****p* < 0.0001, and no significant differences are indicated by “n.s.”.


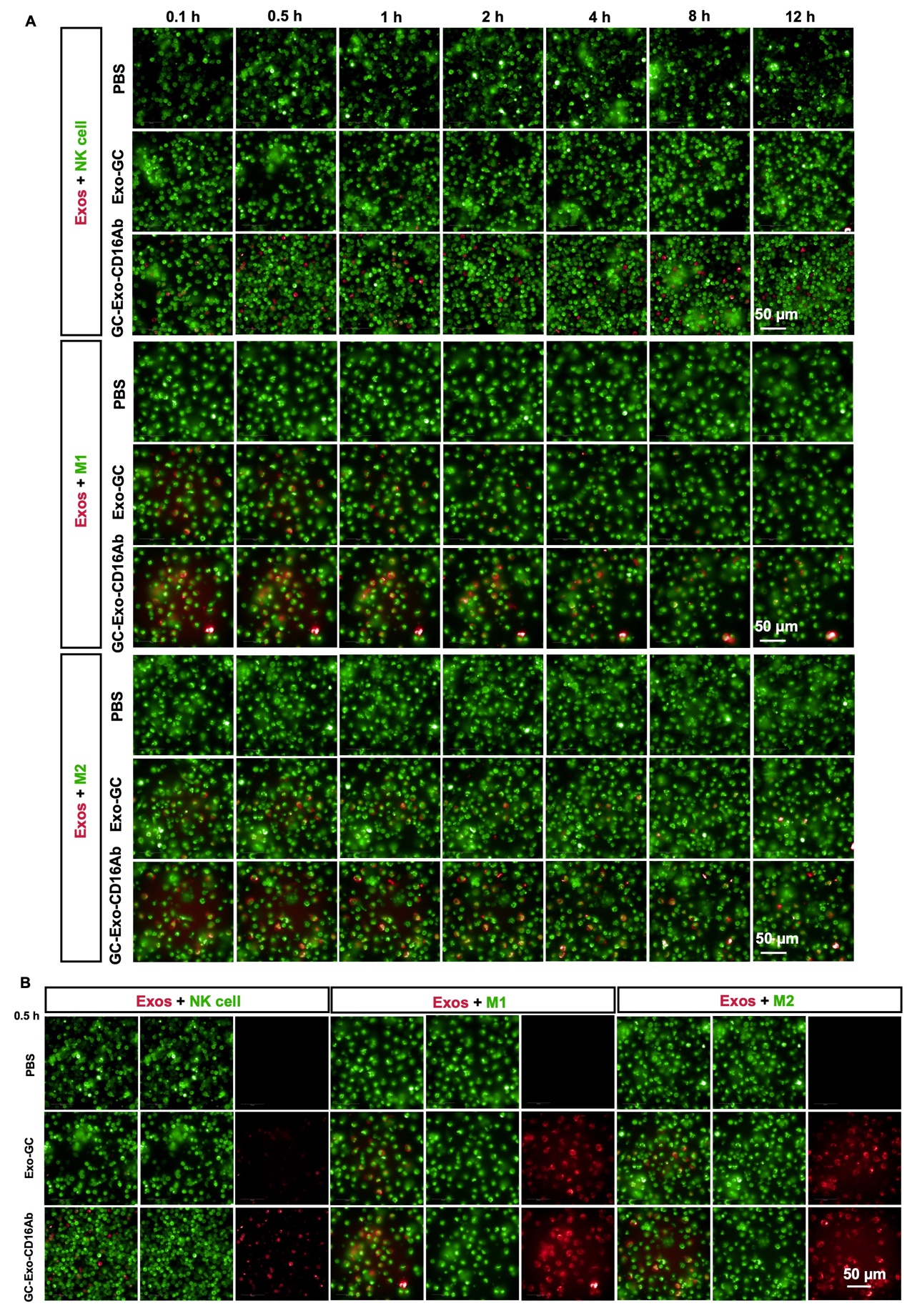


**Figure S4.** Representative IF images of uptake of Exos by NK cells and macrophages. A) The uptake of Exos by NK cells and macrophages with time. B) Representative IF images of uptake of Exos by NK cells and macrophages at 0.5 h. Scale bar, 50 μm.


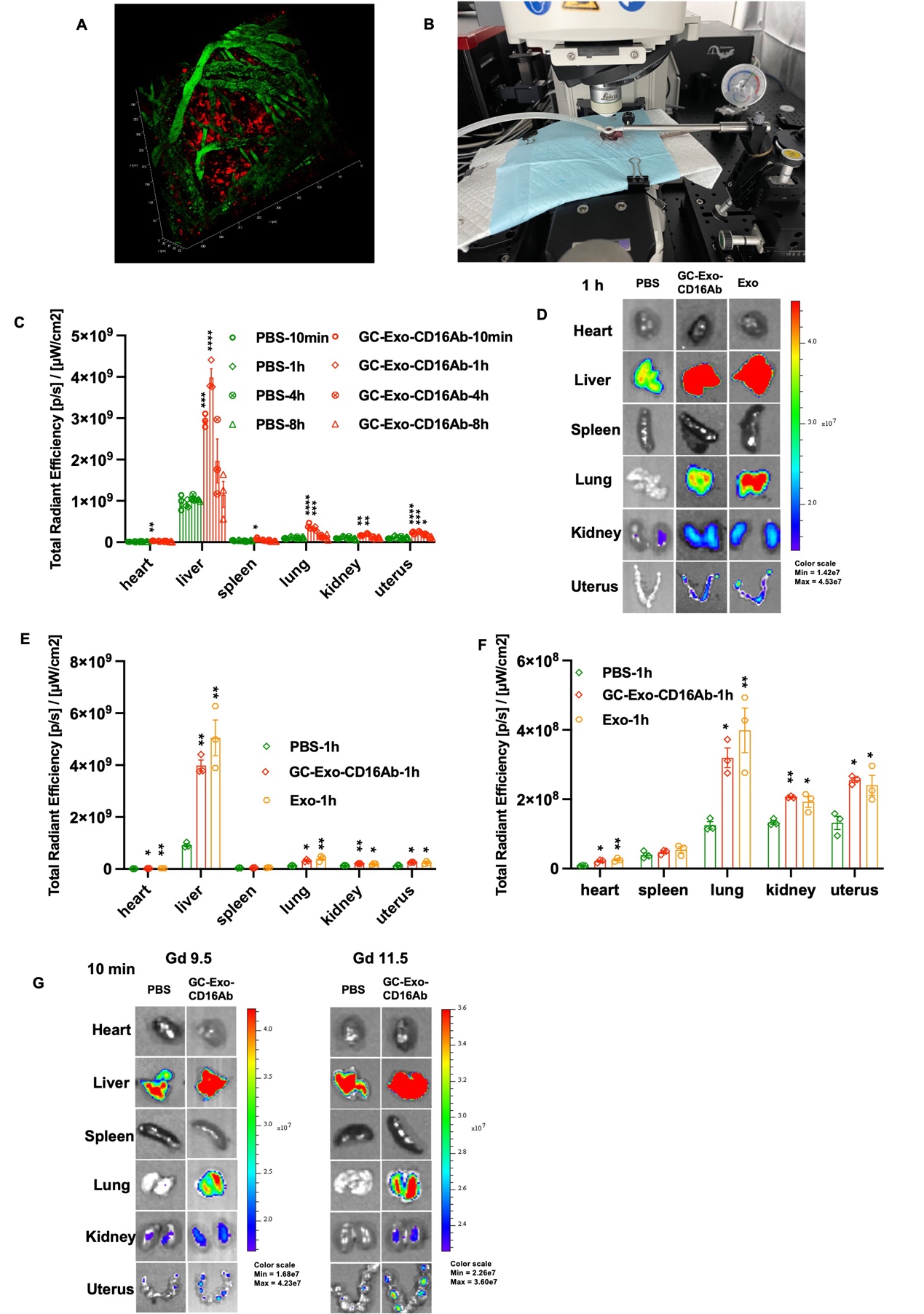


**Figure S5.** The distribution of Exos in vivo. A) Representative 3D image of the distribution of GC-Exo-CD16Ab in the uterus of a pregnancy mouse treated with Exos for 1 h via intravital imaging. Exos were pre-stained with AIE dye (red), and FITC-Dextran (green) was used to indicate vessels. B) The process of intravital imaging. C) Quantitative analysis of the distribution of GC-Exo-CD16Ab in different mouse organs including heart, liver, spleen, lungs, and kidneys. *n* = 3 mice per group. Mice at Gd7.5 were administered Exos dyed with AIE dye through the tail vein. At the specified time points, the organs of mice were isolated and imaged via an IVIS imaging system. D–F) Comparison of the distribution of Exo and GC-Exo-CD16Ab in organs of mice at Gd7.5. Samples were isolated and imaged after being injected with Exos for 1 h. The data on the liver were excluded in (F). *n* = 3 mice per group. G) Representative images of the distribution of Exo-CC-CD16Ab in organs of pregnancy mouse (Gd 9.5 and Gd11.5) treated with Exos for 10 min. One-way ANOVA was used when compared with the PBS groups. **p* < 0.05, ***p* < 0.01, ****p* < 0.001, *****p* < 0.0001.


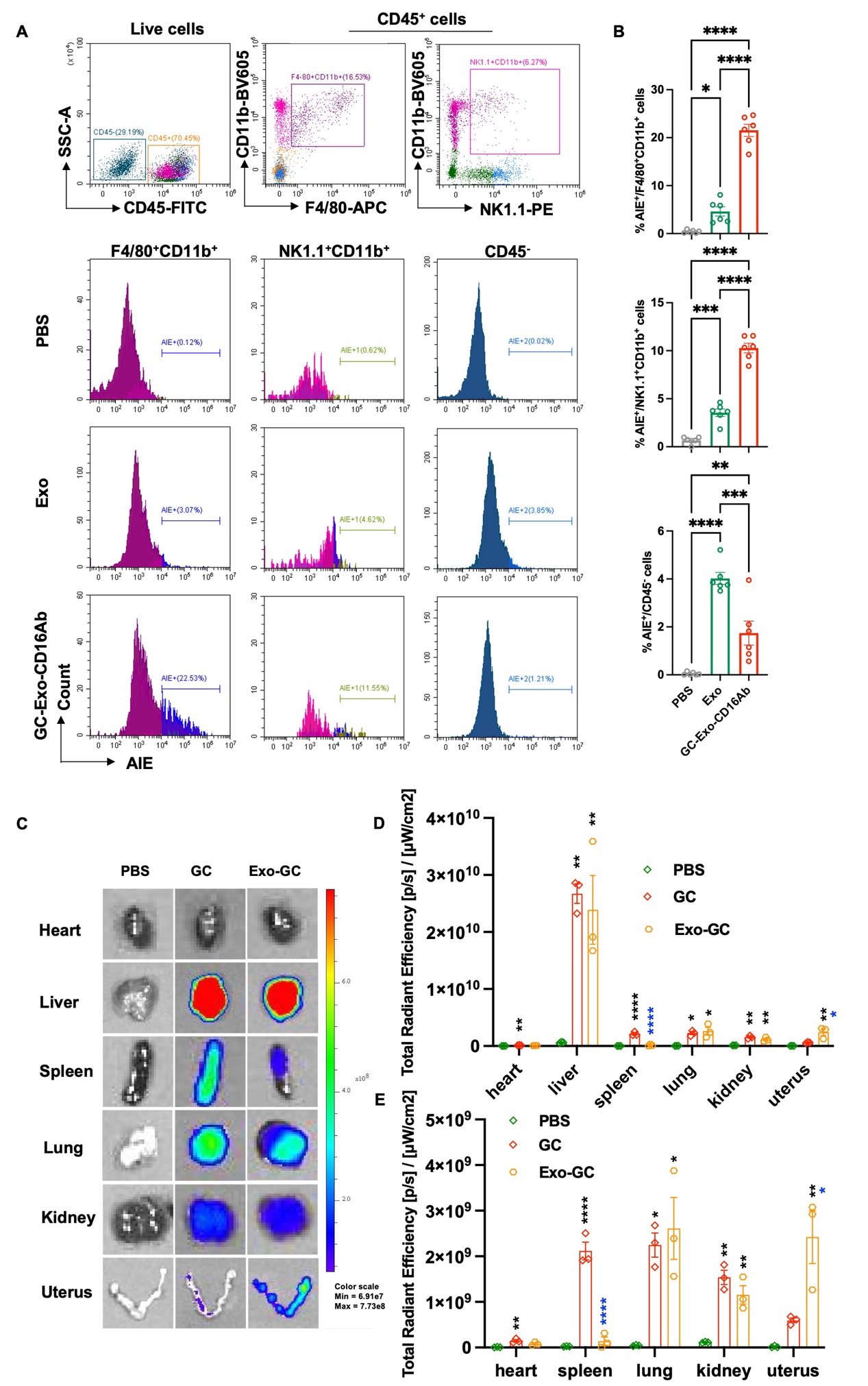


**Figure S6.** The targeting capacity of GC-Exo-CD16Ab and the delivery of GC in vivo. A,B) The percentages of AIE^+^ cells in decidual F4/80^+^CD11b^+^ macrophages, NK1.1^+^CD11b^+^ NK cells, and CD45^-^ cells. *n* = 6 mice per group. C–E) Distribution of GCs in different mouse organs. The data on the liver were excluded in (E). Mice at Gd7.5 were administered with Exos loaded with Cy5.5-GCs through the tail vein. The organs of mice were isolated and imaged via an IVIS imaging system. *n* = 3 mice per group. One-way ANOVA was used (shown as mean ± SEM). D,E) The difference between the PBS group and the other 2 groups is shown with black *, and blue * indicating the difference between the Exo-GC group and the free GC group. **p* < 0.05, ***p* < 0.01, ****p* < 0.001, and *****p* < 0.0001.


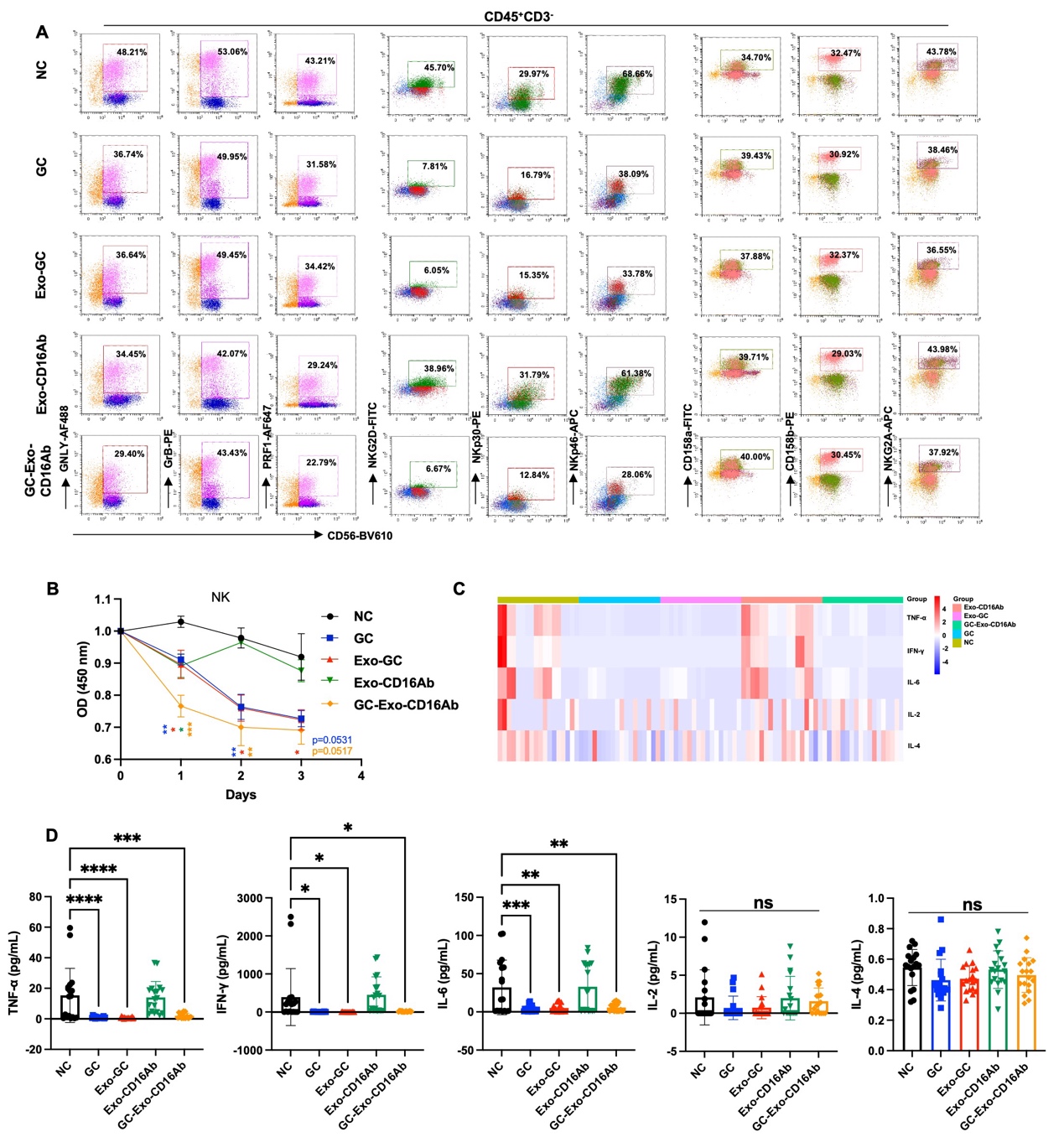


**Figure S7.** GC-Exo-CD16Ab inhibits NK cytotoxicity in vitro. A) Representative FCM images of the percentages of CD56^+^GNLY^+^, CD56^+^CrB^+^, CD56^+^PRF1^+^, CD56^+^NKG2D^+^, CD56^+^NKp30^+^, CD56^+^NKp46^+^, CD56^+^CD158a^+^, CD56^+^CD158b^+^, and CD56^+^NKG2A^+^ NK cells in groups treated with PBS, GC, Exo-GC, Exo-CD16Ab or GC-Exo-CD16Ab for 2 d. B) CCK8 assay of NK cells treated with different GC-Exo-CD16Ab formulations from 0 to 3 d. *n* = 3 independent experiments. C) Heatmap and D) the levels of cytokines in the supernatant of NK cells treated with different GC-Exo-CD16Ab formulations for 2 d. *n* = 18 independent experiments. One-way ANOVA was used when compared with the NC group. All data are represented as mean ± SD. **p* < 0.05, ***p* < 0.01, ****p* < 0.001, *****p* < 0.0001, and no significant differences are indicated by “n.s.”.


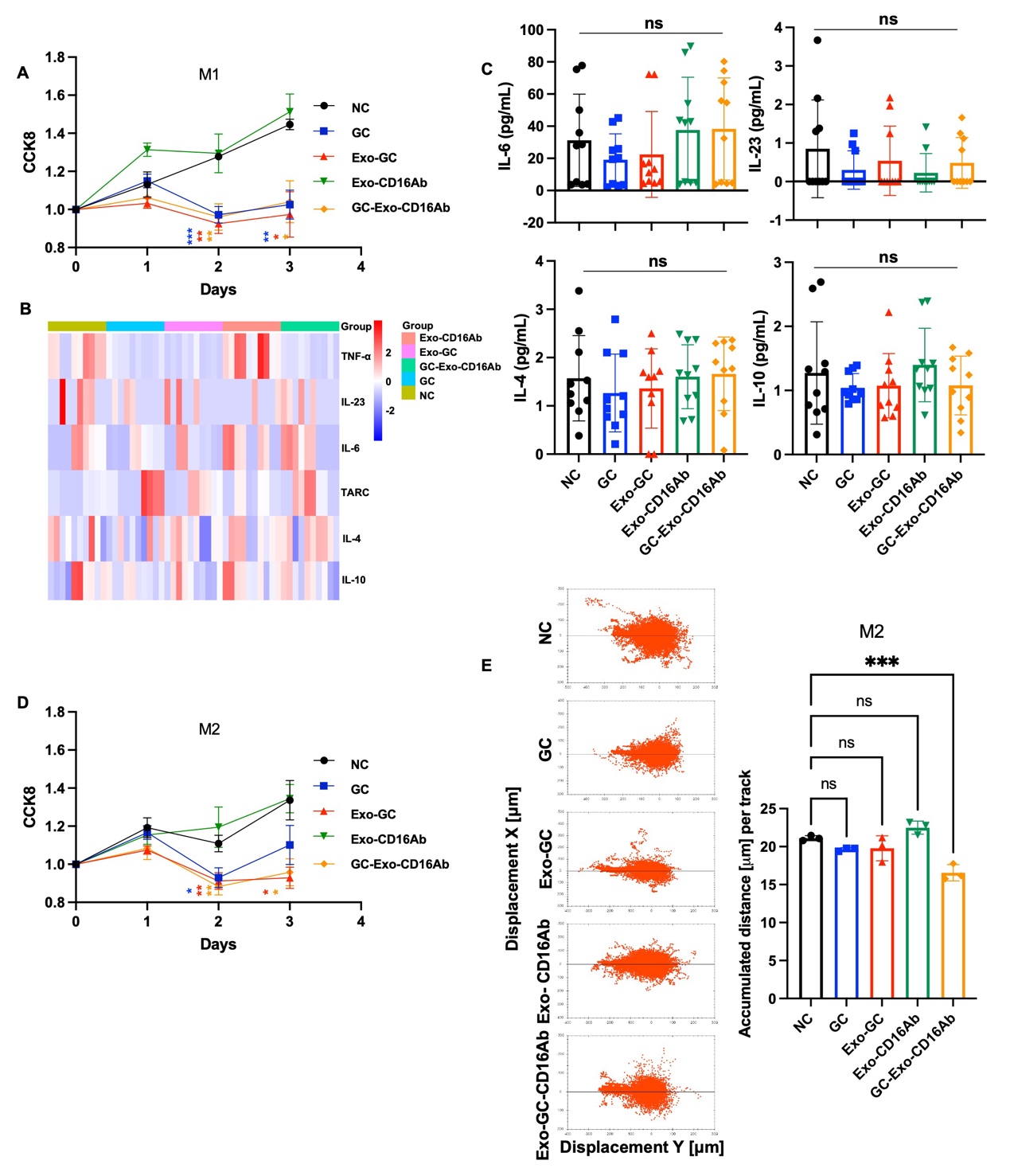


**Figure S8.** GC-Exo-CD16Ab promotes the polarization shift of M1 to M2 macrophages. A) CCK8 assay of M1 macrophages treated with different GC-Exo-CD16Ab formulations from 0 to 3 d. *n* = 3 independent experiments. B) Heatmap and C) the levels of cytokines in the supernatant of M1 macrophages treated with different GC-Exo-CD16Ab formulations for 2 d. *n* = 10 independent experiments. D) CCK8 assay of M2 macrophages treated with different GC-Exo-CD16Ab formulations from 0 to 3 d. *n* = 3 independent experiments. E) Representative images and quantitative analysis of the M2 macrophage trajectory. *n* = 3 independent experiments. One-way ANOVA was used when compared with the NC group. All data are represented as mean ± SD. **p* < 0.05, ***p* < 0.01, ****p* < 0.001, and no significant differences are indicated by “n.s.”.


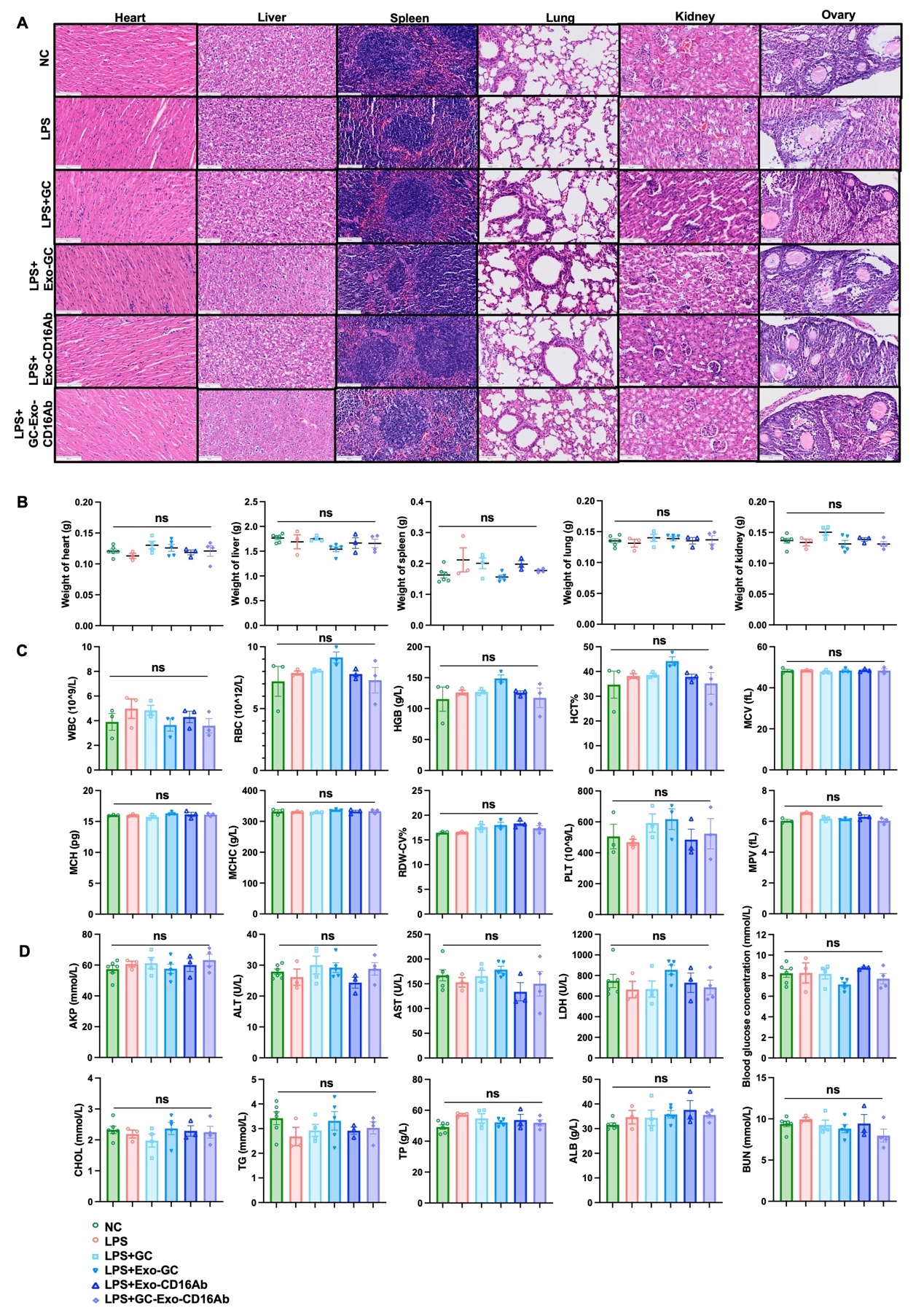


**Figure S9.** Biosafety evaluation of various GC-Exo-CD16Ab formulations. A) H&E staining was used to analyze the inflammatory injury of the heart, liver, spleen, lungs, kidneys, and ovaries in each group (scale bar, 100 μm). B) Organ weight (*n* = 3–6 mice per group), C) routine blood test (*n* = 3 mice per group), and D) blood biochemistry assay (*n* = 3–6 mice per group) were analyzed for each group. One-way ANOVA was used. All data are represented as mean ± SEM. No significant differences are indicated by “n.s.”.


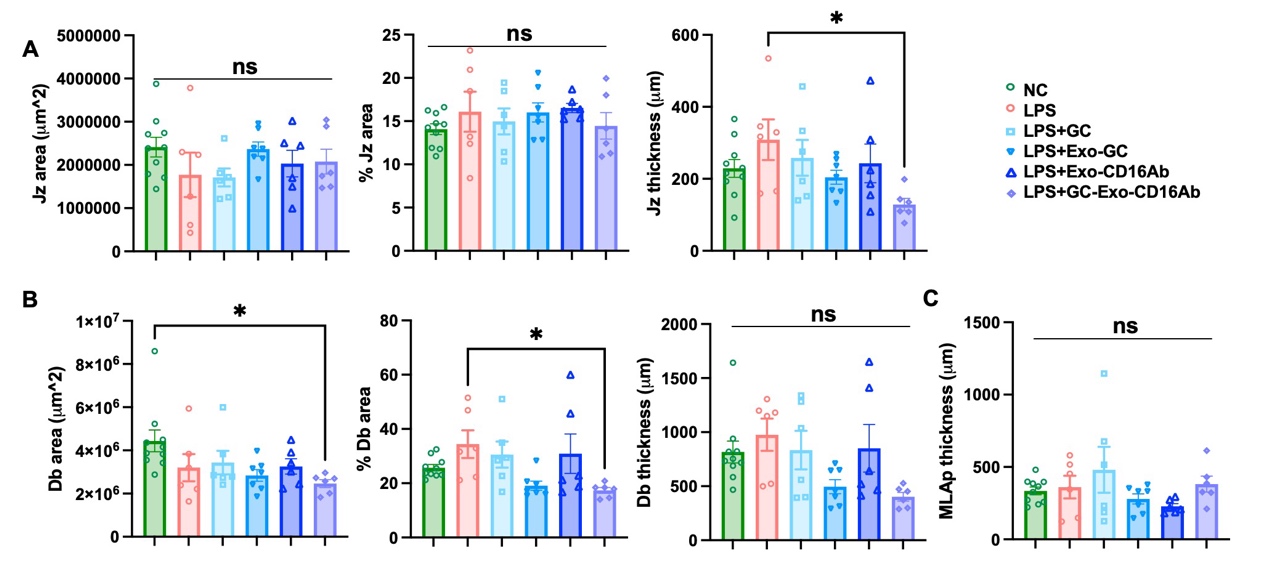


**Figure S10.** A) Area and thickness of junctional zone (Jz), B) mesometrial decidua (basalis, Db), and C) mesometrial lymphoid aggregate of pregnancy (MLAp) in mice injected with various GC-Exo-CD16Ab formulations. *n* = 3–6 mice per group. *n* = 6–10 utero-fetal units per group. One-way ANOVA was used. All data are represented as mean ± SEM. **p* < 0.05, and no significant differences are indicated by “n.s.”.


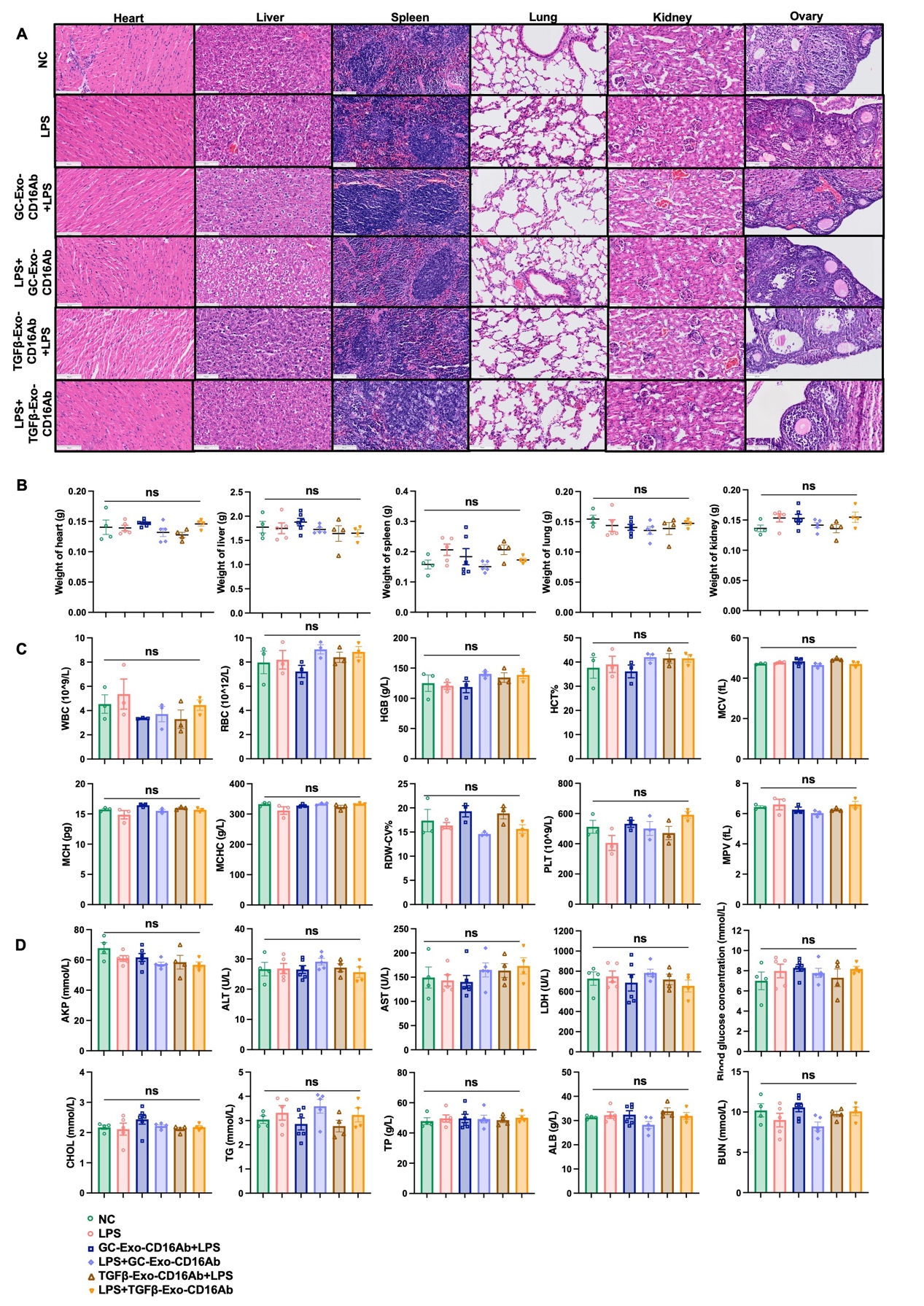


**Figure S11.** Biosafety evaluation of GC-Exo-CD16Ab and TGFβ-Exo-CD16Ab. A) H&E staining was used to analyze the inflammatory injury of the heart, liver, spleen, lungs, kidneys, and ovaries in each group (scale bar, 100 μm). B) Organ weight (*n* = 4–6 mice per group), C) routine blood test (*n* = 3 mice per group), and D) blood biochemistry assay (*n* = 4–6 mice per group) were analyzed for each group. One-way ANOVA was used. All data are represented as mean ± SEM. No significant differences are indicated by “n.s.”.


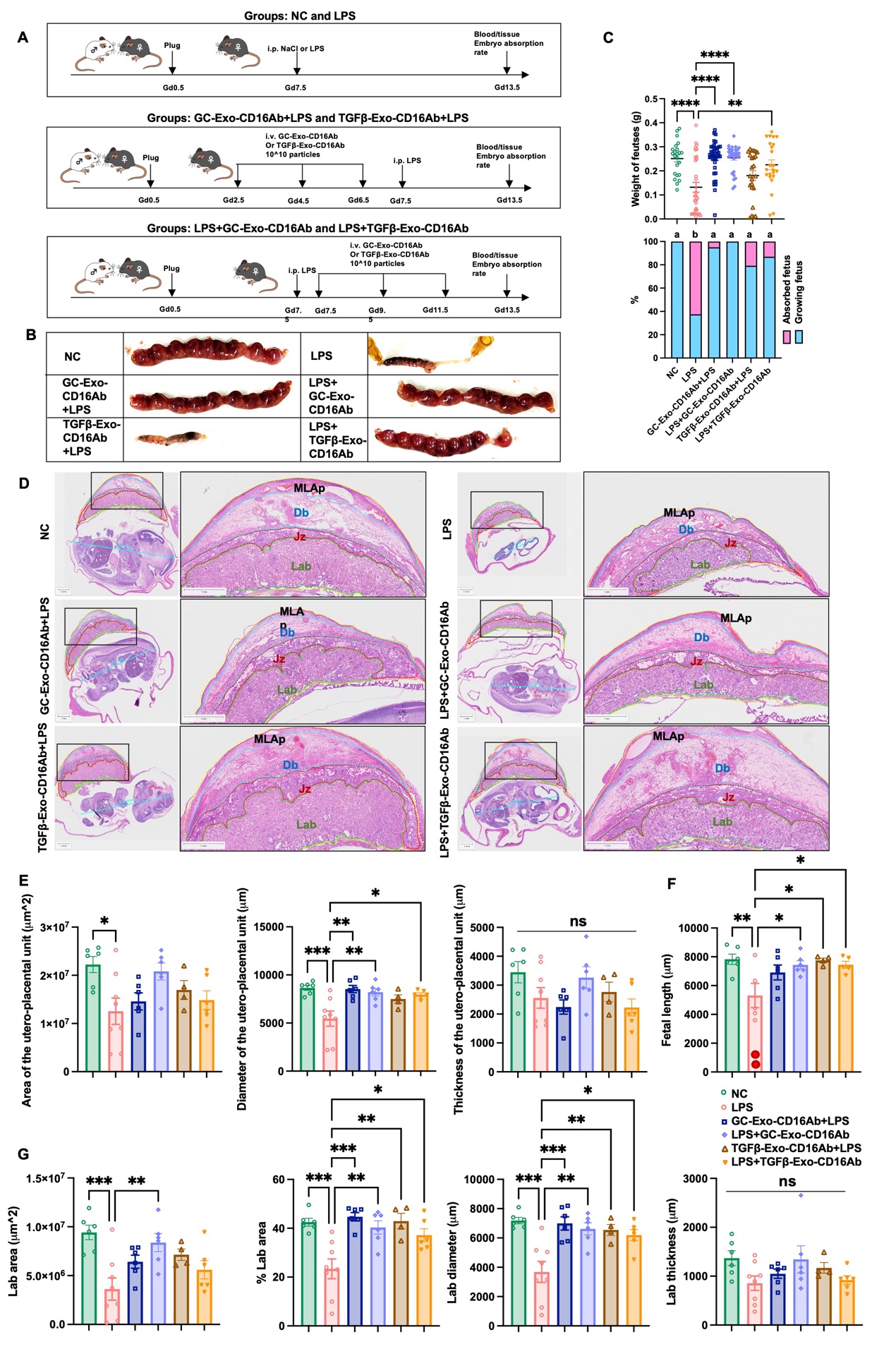


**Figure S12.** GC-Exo-CD16Ab and TGFβ-Exo-CD16Ab promote placental and fetal development, and alleviate abortion. A) Experimental protocol for the therapeutic treatment or prevention of LPS-challenged abortion mice. B) Representative images of pregnancy outcomes in mice injected with GC-Exo-CD16Ab or TGFβ-Exo-CD16Ab. C) Quantitative analysis of fetal weight and the percentages of absorbed fetuses. *n* = 4–6 mice per group. *n* = 23–40 fetuses per group. D) Representative H&E images of the fetus in utero of mice injected with GC-Exo-CD16Ab or TGFβ-Exo-CD16Ab (scale bar, 1 mm). E) Area, diameter, and thickness of the utero-placental unit and G) Lab, and F) fetal length in each group. One red dot represents one absorbed embryo (F). *n* = 4–6 mice per group. *n* = 4–8 utero-fetal units per group. One-way ANOVA was used. All data are represented as mean ± SEM. **p* < 0.05, ***p* < 0.01, ****p* < 0.001, *****p* < 0.0001, and no significant differences are indicated by “n.s.”. Letters a and b were used to show statistically significant differences in the percentages of absorbed fetuses among groups via the chi-squared test.


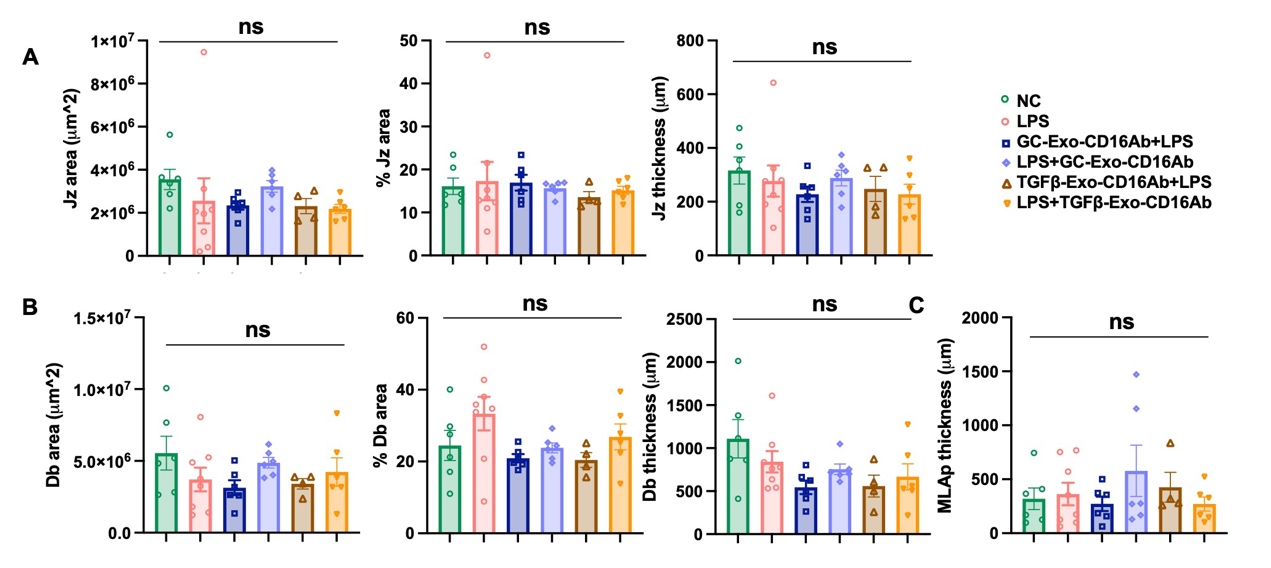


**Figure S13.** A) Area and thickness of Jz, B) Db, and C) MLAp in mice injected with GC-Exo-CD16Ab or TGFβ-Exo-CD16Ab. *n* = 4–6 mice per group. *n* = 4–8 utero-fetal units per group. One-way ANOVA was used. All data are represented as mean ± SEM. No significant differences are indicated by “n.s.”.


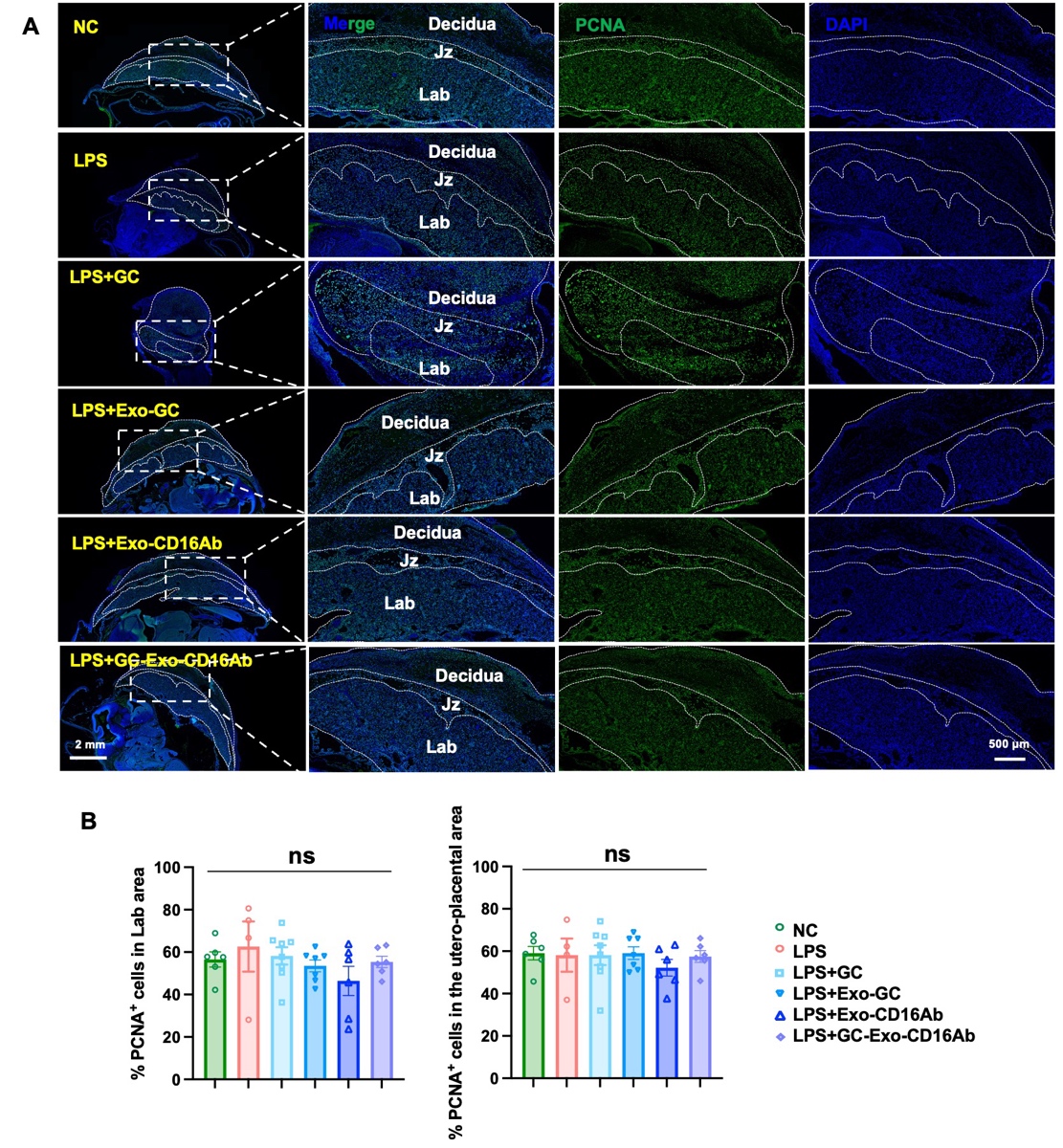


**Figure S14.** A) Representative IHC images and B) quantitative analysis of PCNA^+^ cells in the Lab and in the utero-placental area of mice treated with various GC-Exo-CD16Ab formulations. PCNA: green. Scale bar, 2 mm, 500 μm. *n* = 3–6 mice per group. *n* = 4–8 utero-fetal units per group. No significant differences are indicated by “n.s.”.


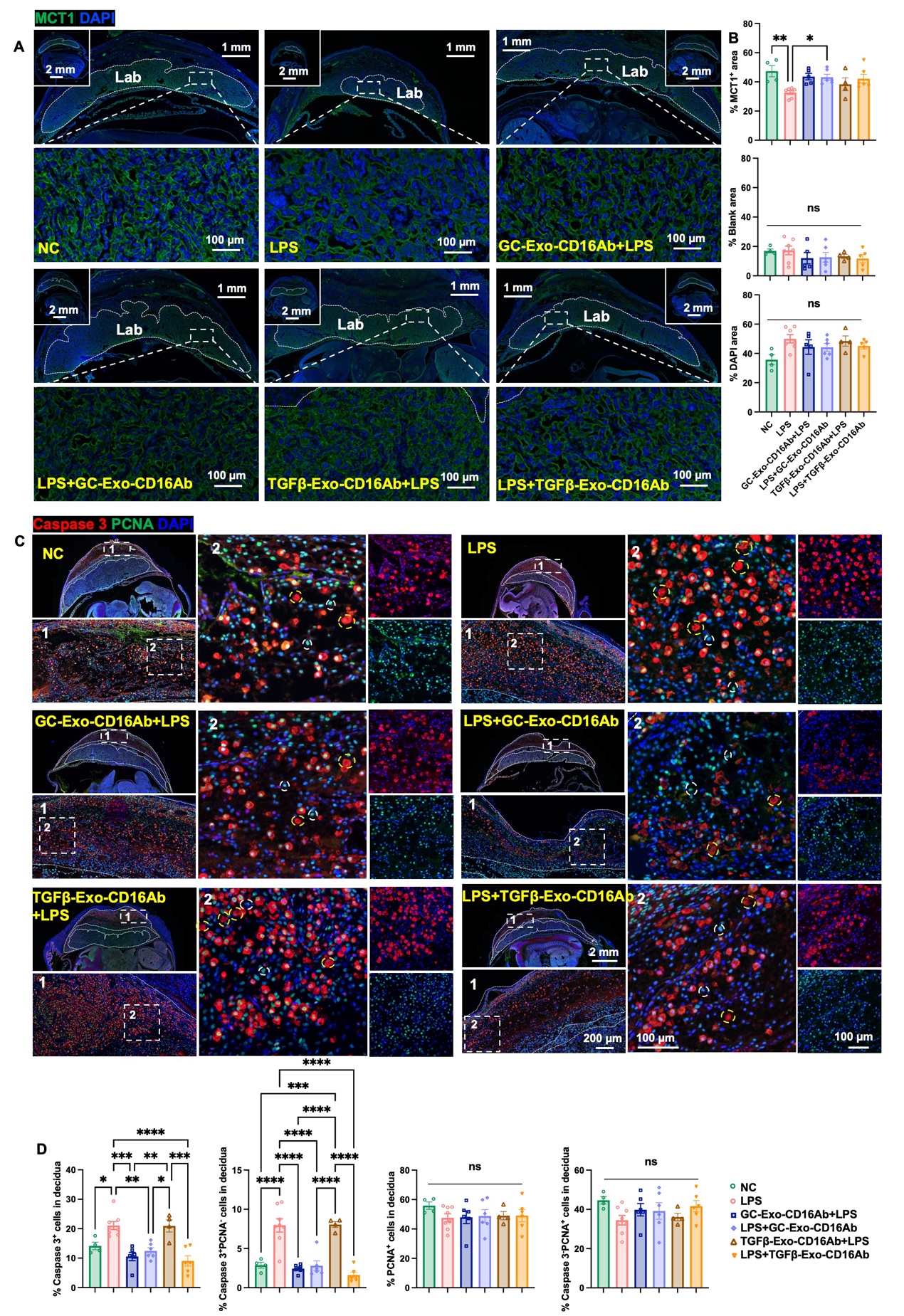


**Figure S15.** Prevention or treatment with GC-Exo-CD16Ab and treatment with TGFβ-Exo-CD16Ab ameliorate structural abnormality of the labyrinth and inhibit apoptosis in decidua in an abortion mouse model. A) Representative IHC images and B) quantitative analysis of placenta stained with MCT1 (green) in each group (scale bar, 2 mm, 1 mm, 100 μm). *n* = 4–6 mice per group. *n* = 4–7 utero-fetal units per group. C) Representative IHC images and D) quantitative analysis of decidual cells stained with Caspase 3 (red) and PCNA (green) (scale bar, 2 mm, 200 μm, 100 μm, 100 μm). White dashed circles indicate Caspase 3^-^PCNA^+^ cells and yellow dashed circles indicate Caspase 3^+^PCNA^-^ cells. *n* = 4–6 mice per group. *n* = 4–8 utero-fetal units per group. One-way ANOVA was used. All data are represented as mean ± SEM. **p* < 0.05, ***p* < 0.01, ****p* < 0.001, *****p* < 0.0001, and no significant differences are indicated by “n.s.”.


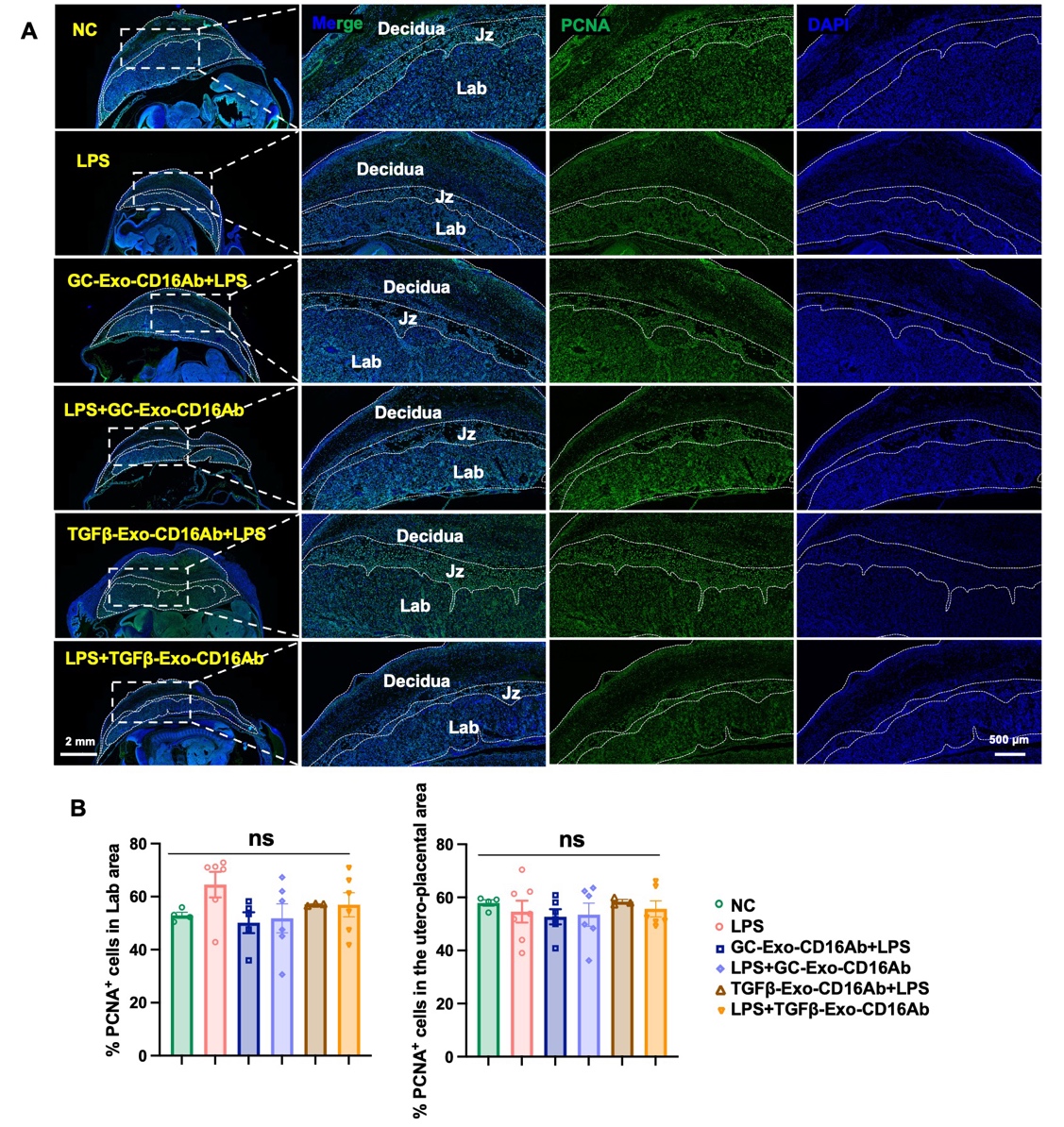


**Figure S16.** A) Representative IHC images and B) quantitative analysis of PCNA^+^ cells in the Lab and the utero-placental area of mice treated with GC-Exo-CD16Ab or TGFβ-Exo-CD16Ab. PCNA: green. Scale bar, 2 mm, 500 μm. *n* = 4–6 mice per group. *n* = 4–6 utero-fetal units per group. No significant differences are indicated by “n.s.”.


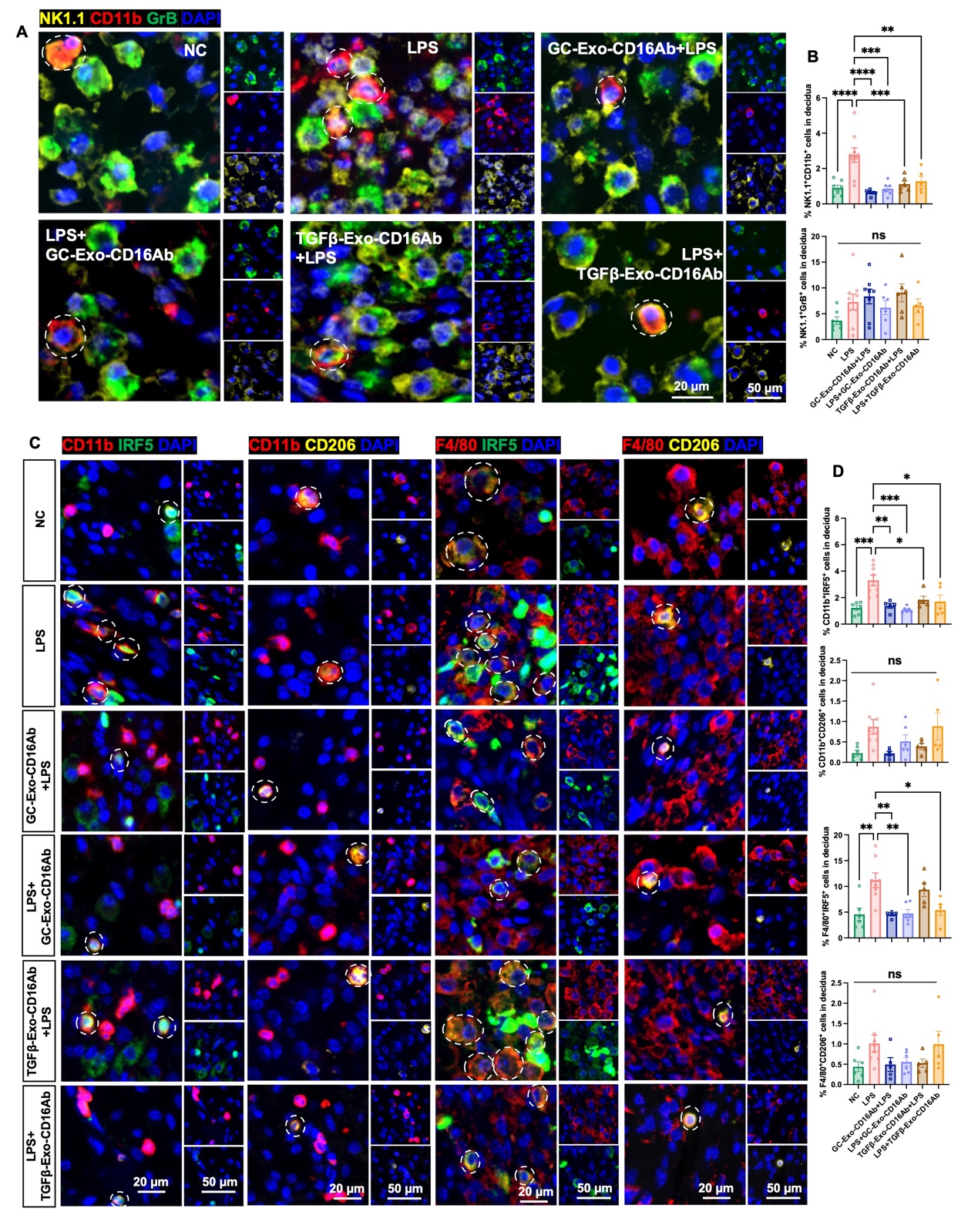


**Figure S17.** GC-Exo-CD16Ab and TGFβ-Exo-CD16Ab regulate immune homeostasis in vivo. A) Representative IHC images and B) quantitative analysis of different NK subsets stained with NK1.1 (yellow), CD11b (red), and GrB (green) in mice (scale bar, 20 and 50 μm). White dashed circles indicate NK1.1^+^CD11b^+^ cells. *n* = 4–6 mice per group. *n* = 5–9 utero-fetal units per group. C) Representative IHC images (indicated with white dashed circles) and D) quantitative analysis of CD11b^+^IRF5^+^, CD11b^+^CD206^+^, F4/80^+^IRF5^+^, and F4/80^+^CD206^+^ macrophages stained with CD11b/F4/80 (red), IRF5 (green), and CD206 (yellow) (scale bar, 20 and 50 μm). *n* = 4–6 mice per group. *n* = 5–8 utero-fetal units per group. One-way ANOVA was used. All data are represented as mean ± SEM. **p* < 0.05, ***p* < 0.01, ****p* < 0.001, *****p* < 0.0001, and no significant differences are indicated by “n.s.”.


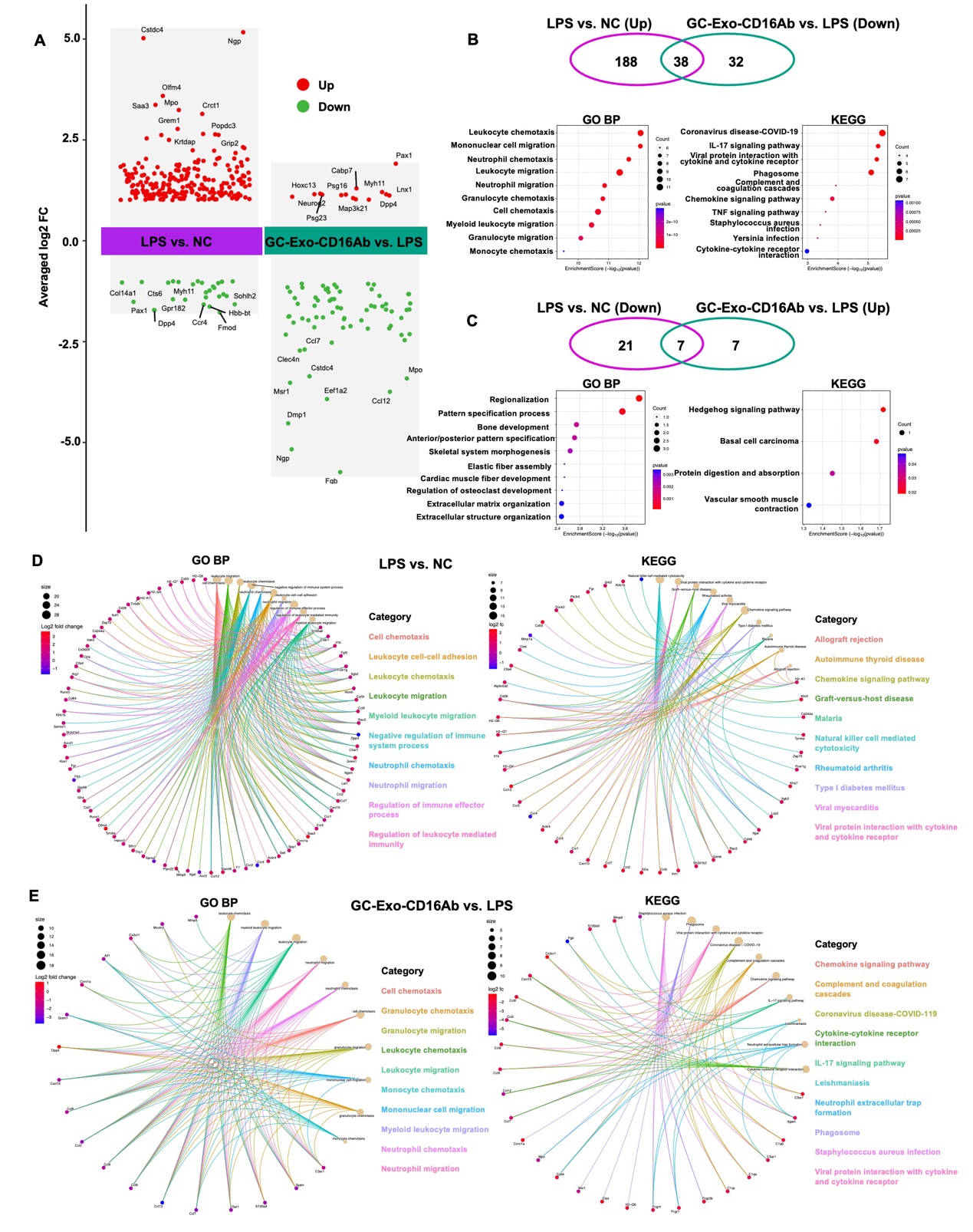


**Figure S18.** GC-Exo-CD16Ab downregulates immune responses caused by LPS treatment in vivo. A) The DEGs in the LPS group compared with the control group, and the DEGs in the GC-Exo-CD16Ab group compared with the LPS group. B,C) GO and KEGG analyses using common DEGs. D,E) GO and KEGG analyses using all DEGs. *n* = 4–5 mice per group.

**
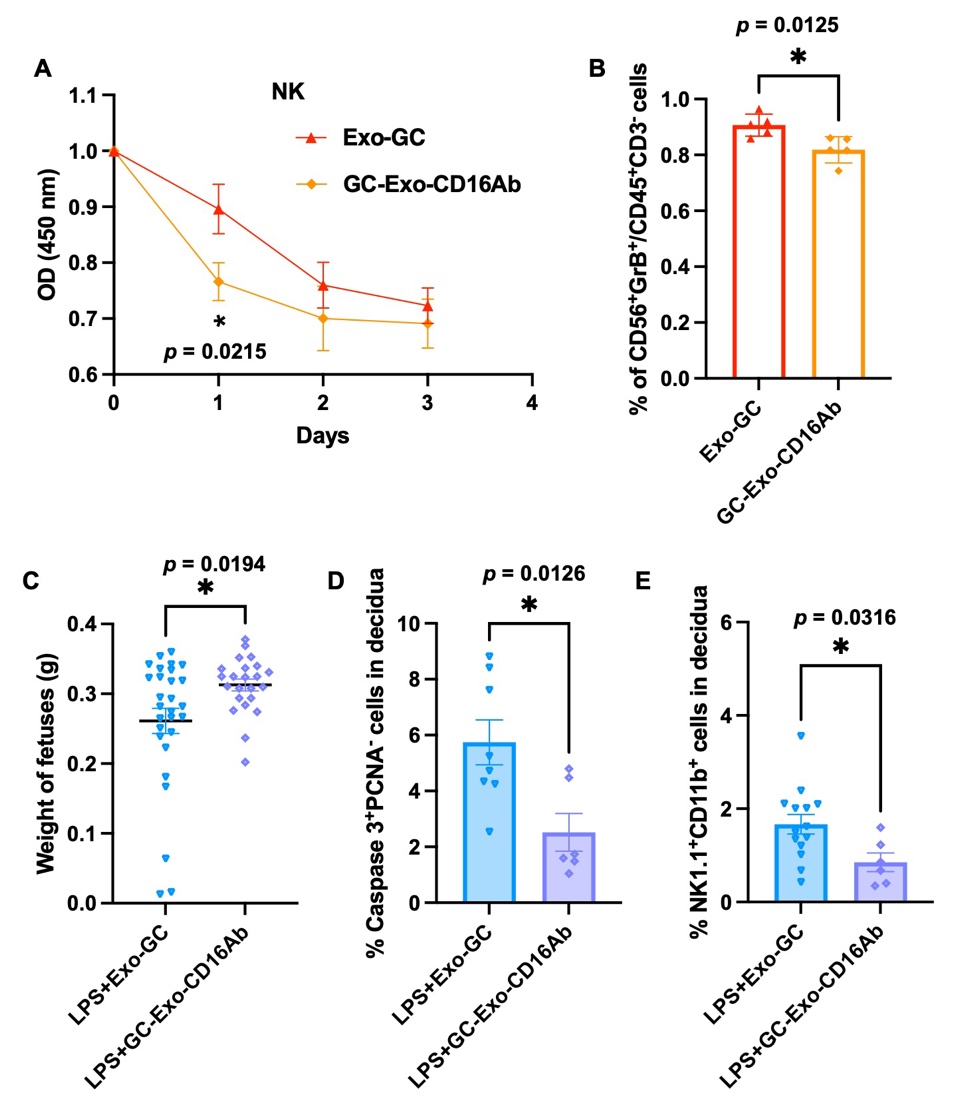
**

**Figure S19.** GC-Exo-CD16Ab has better immunosuppressive and therapeutic effects compared with Exo-GC. A) CCK8 assay of NK cells treated with Exo-GC or GC-Exo-CD16Ab from 0 to 3 d. *n* = 3 independent experiments. B) The percentages of CD56^+^CrB^+^ NK cells. *n* = 5 independent experiments. C) Quantitative analysis of fetal weight. *n* = 4–5 mice per group. *n* = 23–28 fetuses per group. D) The percentage of Caspase 3^+^PCNA^-^ cells in decidua. *n* = 4–5 mice per group. *n* = 6–8 utero-fetal units per group. E) The percentage of NK1.1^+^CD11b^+^ cells in the decidua. *n* = 4–5 mice per group. *n* = 6–14 utero-fetal units per group. An unpaired two-tailed t-test was used. A–C) Data are represented as mean ± SEM. D,E) Data are represented as mean ± SD. **p* < 0.05.
